# An in vitro alveolar macrophage assay for predicting the short-term inhalation toxicity of nanomaterials

**DOI:** 10.1186/s12951-016-0164-2

**Published:** 2016-03-05

**Authors:** Martin Wiemann, Antje Vennemann, Ursula G. Sauer, Karin Wiench, Lan Ma-Hock, Robert Landsiedel

**Affiliations:** IBR R&D gGmbH Institute for Lung Health, Mendelstraße 11, 48149 Münster, Germany; Scientific Consultancy - Animal Welfare, Hallstattfeld 16, 85579 Neubiberg, Germany; BASF SE, Experimental Toxicology and Ecology, GB/TB - Z470, 67056 Ludwigshafen, Germany

**Keywords:** Alveolar macrophages, NR8383 cells, Nanotoxicology, In vitro–in vivo comparison, Inhalation toxicity, 3Rs principle

## Abstract

**Background:**

Most in vitro studies investigating nanomaterial pulmonary toxicity poorly correlate to in vivo inhalation studies. Alveolar macrophages (AMs) play an outstanding role during inhalation exposure since they effectively clear the alveoli from particles. This study addresses the applicability of an in vitro alveolar macrophage assay to distinguish biologically active from passive nanomaterials.

**Methods:**

Rat NR8383 alveolar macrophages were exposed to 18 inorganic nanomaterials, covering AlOOH, BaSO_4_, CeO_2_, Fe_2_O_3_, TiO_2_, ZrO_2_, and ZnO NMs, amorphous SiO_2_ and graphite nanoplatelets, and two nanosized organic pigments. ZrO_2_ and amorphous SiO_2_ were tested without and with surface functionalization. Non-nanosized quartz DQ12 and corundum were used as positive and negative controls, respectively. The test materials were incubated with the cells in protein-free culture medium. Lactate dehydrogenase, glucuronidase, and tumour necrosis factor alpha were assessed after 16 h. In parallel, H_2_O_2_ was assessed after 1.5 h. Using the no-observed-adverse-effect concentrations (NOAECs) from available rat short-term inhalation studies (STIS), the test materials were categorized as active (NOAEC < 10 mg/m^3^) or passive.

**Results:**

*In vitro* data reflected the STIS categorization if a particle surface area-based threshold of <6000 mm^2^/mL was used to determine the biological relevance of the lowest observed significant in vitro effects. Significant effects that were recorded above this threshold were assessed as resulting from test material-unspecific cellular ‘overload’. Test materials were assessed as active if ≥2 of the 4 in vitro parameters undercut this threshold. They were assessed as passive if 0 or 1 parameter was altered. An overall assay accuracy of 95 % was achieved.

**Conclusions:**

The in vitro NR8383 alveolar macrophage assay allows distinguishing active from passive nanomaterials. Thereby, it allows determining whether in vivo short-term inhalation testing is necessary for hazard assessment. Results may also be used to group nanomaterials by biological activity. Further work should aim at validating the assay.

**Electronic supplementary material:**

The online version of this article (doi:10.1186/s12951-016-0164-2) contains supplementary material, which is available to authorized users.

## Background

With the advent of nanotechnology, the importance of adequately assessing the human health impacts of nanomaterials (NMs) is widely recognized. Generally, NMs are covered by chemicals legislation, such as the EU regulation no. 1907/2006 on the registration, evaluation, authorisation and restriction of chemicals (REACH [1]). Taking into account the abundance of NM modifications in regard to particle size, shape, or surface properties and the very broad definition for ‘nanomaterial’ as it has been laid down, e.g., in EU Commission [2], it is expected that the safety of a substantial number of NMs will have to be assessed to meet the REACH information requirements [3]. At the same time, however, the REACH regulation requires that animal testing should only be undertaken as a last resort. Concordantly, Directive 2010/63/EU on the protection of animals used for scientific purposes [4] prescribes implementation of the 3Rs principle to replace, reduce and refine animal testing [5].

To promote the collection of relevant data for the safety assessment of a representative set of NMs, the Organisation for Economic Co-operation and Development Working Party on Manufactured Nanomaterials (OECD WPMN) has launched a Sponsorship Programme for the testing of manufactured nanomaterials [6, 7]. This programme further aims at establishing the role of 3Rs methods for NM testing [6]. With inhalation generally being one of the main routes of NM exposure both for workers and consumers [8, 9], the WPMN has proposed a short-term rat inhalation study (STIS [10]) as a suitable test method to reduce and refine repeated-dose sub-acute inhalation toxicity testing. The STIS provides information on early elements of NM-induced pathogenesis as well as on the reversibility, persistence or progression of effects. Furthermore, NM lung burden and potential for extra-pulmonary translocation may be investigated in the STIS [9–11].

Although the STIS allows reducing animal numbers, suffering, and distress as compared to the 28-day sub-acute inhalation toxicity study described in OECD Test Guideline (TG) 412 [12], it still uses sentient animals. There is also no in vitro test method available that may be used in a tiered approach to decide whether or not in vivo testing, beginning with the STIS, should be required for regulatory hazard assessment. Up to now, numerous different in vitro test systems encompassing submersed or air–liquid exposed cells of mostly pulmonary origin have been investigated in combination with a variety of different endpoint detection methods [13–20]. However, the available in vitro results are inconsistent, and to the best of the authors’ knowledge, there is no in vitro assay that allows reliably predicting the in vivo effects of inhaled NMs [21].

Against this background, it was the goal of the current study to develop an in vitro assay that is suitable for routine regulatory testing of NM inhalation toxicity. In choosing an appropriate test system, it was taken into account which pulmonary cells are predominantly exposed to inhaled NMs. Depending on their aerodynamic diameter, inhaled particles may reach the deep regions of the pulmonary parenchyma [22]. Upon interaction with lung surfactant proteins and lipids, dispersed or agglomerated particles may deposit on the inner alveolar surface. In contrast to the bronchial walls that are protected by a mucus layer which is expelled by the underlying ciliated epithelium, alveolar cells are much more vulnerable. Nevertheless, within the alveoli particles may be engulfed by alveolar macrophages (AMs), which may then be removed via the mucociliary escalator [23–26]. Although nanoparticles were originally believed to be too small to be rapidly cleared by AMs, the dominant role of these cells for the uptake and clearance also of nanoparticles has been shown in various studies [27–29] and is now widely accepted. Only a very small fraction of inhaled NMs appears to permeate the intact lung epithelial barrier thereby potentially becoming systemically available [30–33]. It has also been suggested that nanoparticles may transiently enter the alveolar epithelium or the interstitium, thereby delaying sequestration by AMs [34–36]. Although the full details of the early steps of NM deposition, lung surfactant interaction, and particle transport are still under investigation, it goes undisputed that AMs harbour the major fraction of inhaled NMs, at least after acute or sub-acute exposure [35, 37], and that these cells play a central role in clearing the lung from inhaled NMs.

A multitude of endpoints may be studied to evaluate the biological responses of AMs that are exposed to NMs. AMs are specialized to fend off microbial invasions by non-specific, nicotinamide adenine dinucleotide phosphate-oxidase (NAPDH) oxidase-regulated oxidative burst. Unsurprisingly, also inhaled particles may elicit this type of response, at least upon increased particle load [38, 39]. Formation of extracellular reactive oxygen species (ROS) may elicit indirect oxidative damage of adjacent cells [40]. Moreover, AMs, forming part of the non-specific immune system, may initiate and orchestrate immunological processes by releasing chemokines and cytokines [41]. Together with the formation of extracellular ROS/H_2_O_2_ and/or nitrogen monoxide, AMs are known to release different pro-inflammatory mediators (e.g., tumour necrosis factor α (TNF-α); interleukins (IL-1, IL-6, IL-8); or monocyte chemoattractant protein-1 (MCP-1)) and fibrogenic mediators [e.g., transforming growth factor β (TGF-b); osteopontin; or platelet-derived growth factor (PDGF)] [10, 11, 41–45]. Some of these mediators may serve as chemoattractants for blood granulocytes, or they may enhance pulmonary inflammatory processes [46]. Likewise, AMs may release lytic and other enzymes upon (nano)particle stimulation which in return may activate or stimulate exocytosis [47–49].

Bioactive particles or high particle load may also impair macrophage functions, such as their motility or bactericidal capacity, which may eventually lead to the disruption of cell membrane integrity [23, 24, 50–52]. Although AMs are readily recruited from blood monocytes, any weakening of the intra-pulmonary AM population will affect lung clearance thereby enhancing the risk for the evolvement of lung diseases [53].

Since the pulmonary effects of NMs upon short-term inhalation exposure are predominantly AM-mediated, it appears promising to study essential biological responses of AMs in vitro when aiming at predicting in vivo short-term inhalation effects. This principle was established long before the term nanotoxicology was coined. Rehn et al. [54, 55] incubated a defined number of primary AMs lavaged from the lungs of guinea pigs and rats with a defined mass of micron-scaled particulate matter, such that a mean particle burden/AM could be calculated. Rehn et al. [55] designed their in vitro studies to cover the plausible in vivo particle burden/AM inside the rat lung. Assuming that the upper in vitro threshold corresponds to the upper in vivo rat lung threshold since primary rat AMs widely resemble in vivo rat AMs and these cells sequester the vast majority of inhaled particles, the relevant concentration range was estimated from the maximum rat lung burden divided by the number of AMs/lung. This number is fairly constant (i.e., 1–2 × 10^7^) in healthy, unrestrained rats [55, 56]. Accordingly, Bruch et al. [57] calculated a mean particle burden of ≤120 pg/AM as a realistic in vivo upper value. Interestingly, direct measurements carried out on AMs extracted from the bronchoalveolar lavage fluid (BALF) of rats exposed to 30 mg/m^3^ poorly soluble AlOOH NMs by inhalation for 28 days revealed a cell burden of approx. 90 pg/AM [58]. Altogether, these findings underline that cultured AMs may be exposed to doses that resemble in vivo conditions. This is a promising starting point for in vitro-in vivo comparisons.

An in vitro assay suitable for regulatory toxicity testing should include biologically relevant, but easy to measure endpoints. This will increase the predictive value of the in vitro data. The model originally proposed by Rehn et al. [55] comprised TNF-α (as a major pro-inflammatory cytokine), ROS/H_2_O_2_ (as a major inducer of oxidative stress), β-glucuronidase (GLU; indicating macrophage activation [44] and/or membrane damage), and AM bactericidal capacity (revealing changes in the viability of the particle-laden AMs). Since these four endpoints (or parameters) were visualized as four vectors, the in vitro assay was termed ‘vector model’. The ‘vector model’ has been used to assess the in vitro effects of different materials relevant for the occupational setting [50, 57, 59].

In the present study, the concept of the ‘vector model’ was transferred to NR8383 cells, an AM cell line derived from rat lung lavage cells [60, 61]. Over many passages, NR8383 cells maintain their typical AM-like size, appearance, and phagocytic as well as immunological properties. They have been observed to react to test material exposure by the formation and release of different pro-inflammatory and fibrogenic cytokines and chemokines, including TNF-α, IL-1, TGF-β and PDFG [60–65]. NR8383 cells have further been used to measure oxidative burst and ROS production as well as mitochondrial damage [61, 66]. On the gene expression level, different markers for oxidative damage, inducible nitric oxide synthase expression [64], inflammation, autophagy, and apoptotic balance were recorded in NR8383 cells [67].

Just as macrophages in general [68], NR8383 cells are more sensitive to test materials than e.g., lung epithelial cells [69]. NR8383 have been used for the in vitro testing of a variety of NMs including functionalized amorphous SiO_2_, indium tin oxide, alumina, Al_2_O_3_, ultrafine TiO_2_, (multi-walled) carbon nanotubes ((MW)CNTs), various copolymers and also heparin nanoparticles [64, 66, 69–75]. Generally, these studies differed with respect to fundamental aspects of the test protocol, such as medium composition, size of culture vessels, cell density, or incubation period. Furthermore, oftentimes well established materials serving as negative or positive controls (NCs, PCs) were not included. This, however, is a mandatory prerequisite for regulatory testing. It further forms an essential part of benchmark in vitro testing. Benchmark testing implies the comparative assessment of new materials against ‘benchmark materials’ which were previously tested and evaluated according to standard criteria. This benchmark testing is expected to constitute an important pillar in the safety assessment of the abundance of NM modifications available [21, 76].

In the present study, a test protocol was set up and applied that largely followed up on the testing strategy of the ‘vector model’ published by Bruch et al. [57, 77, 78], concordantly using non-nanosized corundum (Al_2_O_3_) and quartz DQ12 as NC and PC, respectively. Corundum was further used as negative (albeit micron-scaled) benchmark material, against which the in vitro test data were compared. The permanent NR8383 cells were selected as test system, and test materials were applied under protein-free cell culture conditions since protein supplementation of the culture medium has been observed to mitigate the in vitro cellular effects of NMs [21]. The selected endpoints comprised NM effects on membrane disruption and macrophage activation that were assessed by measuring cellular release of lactate dehydrogenase (LDH) and GLU. Pro-inflammatory reactions were assessed by measuring cellular release of bioactive TNF-α, and induction of oxidative stress was assessed by measuring the formation and release of H_2_O_2_.

Using this testing strategy, 18 inorganic NMs were assessed, covering metal oxides and sulphates (AlOOH, BaSO_4_, CeO_2_, Fe_2_O_3_, TiO_2_, ZnO, and ZrO_2_), precipitated, pyrogenic and colloidal amorphous SiO_2_ and graphite nanoplatelets, and two nanosized organic pigments. CeO_2_, ZrO_2_ and colloidal amorphous SiO_2_ were tested without and with doping or surface functionalization. Thereby, the selected test materials covered a broad range of chemically different, but economically important nanosized materials.

The in vitro data collected performing the NR8383 AM assay evaluating LDH, GLU, TNF-α and H_2_O_2_ release were used to obtain an overview on the cellular effects of the altogether 20 test materials. Moreover, the present study was conceived to assess the applicability of the in vitro NR8383 AM assay within a tiered approach for regulatory NM hazard assessment. An example for such a tiered approach is the DF4nanoGrouping Decision-making framework for the grouping and testing of NMs put forward by Arts et al. [33, 79]. Within a tiered approach, the information gathered during the early non-animal tiers is used to determine whether higher tier in vivo testing is relevant for hazard assessment, or not.

Accordingly, it was assessed whether the data from the in vitro NR8383 assay allow recognizing passive NMs that will not elicit specific toxic effects upon inhalation exposure. If a NM can be assigned to the group of passive NMs based on the outcome of the in vitro NR8383 AM assay along with material and functional properties, in vivo inhalation studies may not be required for its hazard assessment. Further, it was assessed if the in vitro NR8383 AM assay allows identifying active NMs which elicit specific toxic effects upon inhalation exposure. Active NMs require further data and sub-grouping using in vivo inhalation testing to facilitate their hazard assessment.

Against this background, the in vitro data were used to develop a prediction model for the in vitro NR8383 AM assay that appeared best suited to distinguish passive from active NMs. As a ‘gold standard’ for the categorization of active and passive NMs, the findings from STISs (or sub-acute inhalation studies) available for all 20 test materials [10, 11, 58, 79–82] were used. A rat STIS no-observed-adverse-effect concentration (NOAEC) of <10 mg/m^3^ was set as threshold value indicating NM activity, whereas NMs with STIS NOAECs of 10 mg/m^3^ or higher were assessed as passive [33, 79]. Applicability of the in vitro NR8383 AM assay within a tiered approach for regulatory hazard assessment was evaluated by comparing the in vitro assignments of the test materials as either active or passive to the in vivo categorization.

## Methods

### Test materials

Ten of the altogether 18 inorganic NMs of the present study were used in the German Federal Ministry for Education and Research-funded projects NanoCare (i.e., AlOOH, BaSO_4_ NM-220, nano-CeO_2_, Al-doped CeO_2_) and NanoGEM (i.e., ZrO_2_.TODA, ZrO_2_.acrylate, colloidal SiO_2_.naked (Levasil^®^ 200) and its surface-functionalized variants SiO_2_.PEG, SiO_2_.amino, SiO_2_.phosphate). Details on their physico-chemical characterization have been published by Driessen et al. [83], Kroll et al. [17], Kuhlbusch et al. [84], Hellack et al. [85], Izak-Nau and Voetz [86], and Landsiedel et al. [11].

Of these test materials, AlOOH (boehmite) was originally supplied by Sasol (Germany); BaSO_4_ NM-220 by Solvay (Belgium); Al-doped CeO_2_ by Evonik Industries AG (Germany); ZrO_2_.TODA and ZrO_2_.acrylate by itN Nanovation AG (Germany); SiO_2_.naked by AkzoNobel AB (Sweden); and nano-CeO_2_, SiO_2_.PEG, SiO_2_.amino, and SiO_2_.phosphate by BASF SE (Germany).

Just as BaSO_4_ NM-220, also six further NMs (i.e., TiO_2_ NM-105, ZnO NM-111, precipitated SiO_2_ NM-200 and pyrogenic SiO_2_ NM-203, CeO_2_ NM-211 and CeO_2_ NM-212) were representative NMs of the OECD WPMN Sponsorship Programme for the Testing of Manufactured Nanomaterials. These six NMs were obtained from the EU Commission’s Joint Research Centre (JRC, Ispra, Italy). Detailed characterization data for these OECD representative NMs have been published by Singh et al. [87, 88] and Rasmussen et al. [89, 90]. Of note, NM-x numberings (e.g., ZnO NM-110) refer to the respective codes of the OECD representative NMs (http://www.oecd.org/science/nanosafety/).

The inorganic red pigment Fe_2_O_3_ (hematite), the two nanosized organic pigments Diketopyrrololpyrrol (DPP) Orange N, and Pigment Blue 15:1 (Cu-phthalocyanin) and graphite nanoplatelets (GraphEx^®^) were provided by BASF SE, Germany (*cf.* Arts et al. [79] for extensive characterization data).

Finally, the micron-sized NC and benchmark material corundum (d_50_: 2.2 μm) was purchased from ESK (Elektroschmelzwerk Kempten, Germany) and the micron-sized PC quartz dust DQ12 (d_50_: 2.1 μm) from DMT GmbH & Co. KG (Germany).

### Preparation of test material suspensions

All test materials were suspended in the cell culture medium (F-12K; Biochrom GmbH, Germany) and, for H_2_O_2_ determination, in Krebs-Ringer phosphate glucose (KRPG) buffer. KRPG buffer (pH value 7.3–7.4) is a physiological salt solution that contains 129 mM NaCl, 4.86 mM KCl, 1.22 mM CaCl_2_, 15.8 mM NaH_2_PO_4_, and 5.5 mM glucose. In preparing the test material suspensions, approx. 1 mg of the dry-powder test items was weighed into a 15 mL polypropylene tube (Greiner, Germany). The necessary amount of fluid (3–10 mL) was added to achieve final maximum concentrations of 180 μg/mL F-12K medium or 360 μg/mL KRPG buffer, the latter being diluted twofold under testing conditions. Test items delivered as liquid suspensions (i.e., SiO_2_.naked and its surface functionalized variants and both ZrO_2_ NMs) were diluted accordingly. Upon suspension and/or dilution, the tubes were briefly vortexed to remove particles from the vessel wall. Afterwards, the preparations were ultrasonicated for 10 s using a probe adjusted to 50 W (Vibra Cell™, Sonics & Materials, USA). This step was recognized as indispensable to separate aggregates of the micron-scaled controls quartz DQ12 and corundum, and it was also included in preparing the dry-powder and liquid test items.

Due to poor dispersibility, the general protocol for test material preparation was adapted for the following test materials: SiO_2_ NM-200 and NM-203, which were delivered strongly agglomerated, were treated with an ultrasonic probe on ice (5× for 1 min, each). Pigment Blue 15:1 and graphite nanoplatelets required pre-wetting in 0.5 % (v/v) ethanol. For this purpose, a stock dispersion of 2.56 mg/mL (with the further addition of 0.05 % (w/v) bovine serum albumin in the case of Pigment Blue 15:1 to warrant dispersion) was prepared by 10-min bath ultrasonication (Sonorex, DT106, Bandelin Electronic, Berlin, Germany). Fe_2_O_3_ and DPP Orange N were bath ultrasonicated (2× for 10 min, each) upon dispersion in double distilled water.

All test material suspensions were prepared shortly before in vitro application. For LDH, GLU and TNF-α determination, the suspensions were serially diluted with F-12K medium to achieve test concentrations of 22.5, 45, 90, and 180 μg/mL. For H_2_O_2_ determination, the test material suspensions were first diluted in KRPG buffer to a twofold of these concentrations (i.e., 45–360 μg/mL) since the Amplex red assay begins with a 1:1 dilution step (*cf.* “H_2_O_2_ formation” section). Accordingly, all four endpoints were generally assessed at final test material concentrations of 22.5-180 μg/mL. As exceptions from this rule, the highly bioactive ZnO NM-111 was tested for LDH and TNF-α at 2.8, 5.6, 11.2 and 22.5 μg/mL (but up to 180 μg/ml for GLU and H_2_O_2_). For technical reasons, ZrO_2_.acrylate was assessed at 35–283 μg/mL. For all tests, material-free vehicle controls were carried out.

### Particle size distribution in water, F-12K medium or KRPG buffer

The particle size distribution and agglomerating properties of the test materials suspended in water were assessed by particle tracking in the supernatant (detection range: 30–1000 nm) and by inspection of the agglomerates (detection range: >0.7 μm). All test material suspensions were diluted as necessary to achieve optimal measurement conditions of 5 × 10^8^ particles/mL. Laser illumination combined with tracking analysis allowed detecting nanoparticles down to a concentration of 1 × 10^8^/mL: The size distribution of the dispersed particles was evaluated with a NanoSight LM 10 instrument (Malvern Instruments, England) equipped with a green (532 nm) or blue (405 nm) laser and using the accompanying NTA software 2.1–2.3. Agglomerated and gravitationally settled particles were recorded at the bottom of the culture vessels (96 well plates) using an inverted microscope (Axiocert 40C, Zeiss, Germany) equipped with phase contrast optics (10× or 20× objectives). Digital images (Axiocam C3, Zeiss, Germany) were taken after 16 h in the absence (cell-free controls) or presence of cells to document particle uptake under testing conditions [91].

Additionally, the test materials were analysed as described above suspended in 360 μg/mL KRPG buffer or 180 μg/mL F-12K medium. The incubation conditions were identical to the ones applied during in vitro testing, apart from the fact that no NR8383 cells were provided. Again, the suspensions were diluted as necessary to achieve optimal measurement conditions.

### Preparation of the NR8383 test system

Rat NR8383 cells [60, 61] were originally purchased from ATCC (USA) and cultured in F-12K medium supplemented with 2 mM glutamine, penicillin/streptomycin (100 U/10 mg/mL; and 15 % (v/v) fetal calf serum (FCS; all from PAN Biotech GmbH, Germany). Cells were grown in 500 mL flasks (Greiner, Germany) under standard cell culture conditions (37 °C; 5 % CO_2_) and passaged once a week. For the in vitro tests, cells were detached from the substrate by mechanical agitation, dispersed by pipetting, seeded into 96-well plates at 3 × 10^5^ live cells per well and incubated in F-12K medium supplemented with 5 % FCS for 24 h. For test material application, supernatants were withdrawn, and test material-containing phenol red-free F-12K medium (Biochrom GmbH, Germany), supplemented with 2 mM glutamine and 100 U/100 μg/mL penicillin/streptomycin, was applied onto the cells. To correct for test material-specific adsorption and/or scattering of light, cell-free NM-containing controls were included in all test runs for all dilution steps. Cells were incubated with particles for 16 or 1.5 h. For the determination of LDH, GLU, and TNF-α release, cell culture supernatants were sampled after 16 h of incubation. In a parallel approach, supernatants were sampled after 1.5 h of incubation to assess H_2_O_2_ formation.

### Lactate dehydrogenase and glucuronidase

To measure the amount of LDH and GLU released from the treated NR8383 cells, cell culture supernatants were harvested and centrifuged (10 min, 200 g) to remove cell debris. From each well, 50 μL was incubated with LDH reaction mix (Roche Cytotoxicity Kit; Roche, Germany) and evaluated as described by the manufacturer. Measurements were corrected for cell free-adsorption and normalized to the PC value (set to 100 %) obtained from NR8383 cells lysed with 0.1 % Triton X-100 (Sigma Aldrich, Germany). To measure GLU activity, 50 µL of the supernatant was incubated at 37 °C with 100 μL 0.2 M sodium acetate buffer (pH 5) containing 13.3 mM p-nitrophenyl-d-glucuronide (Sigma Aldrich, Germany) and 0.1 % Triton X-100 [50]. The reaction was terminated after 2 h by addition of 100 μL 0.2 M NaOH (Merck KGaA, Germany). Optical density (OD) was measured at 405 nm in a plate photometer (Tecan 200Pro, Tecan, Germany); values were corrected for cell free-adsorption and normalized to the PC value (set to 100 %) again obtained from NR8383 cells lysed with 0.1 % Triton X-100.

### Bioactive tumour necrosis factor alpha

To measure the bioactive TNF-α released from the treated NR8383 cells, cell culture supernatants were centrifuged and analysed using the L929 cytolysis test described by Desch et al. [92]. Briefly, 50 μL supernatant was pipetted onto 80 % confluent L929 mouse fibroblasts (ATCC, USA) in the presence of actinomycin D (Sigma Aldrich, Germany). After 24 h, the L929 cells were washed with phosphate buffered saline (PBS; Biochrom GmbH, Germany), stained with 0.5 % crystal violet (Sigma Aldrich, Germany), washed extensively with PBS, and lysed in an acidic mixture of citrate-buffered 50 % ethanol (Carl Roth GmbH, Germany). OD was measured at 570 nm, and results were expressed as L929 fibroblast lysis relative to non-treated medium controls, which were set to 0 %. Additionally, the L929 cells’ responsiveness to TNF-α was controlled using a TNF-α standard (510-RT; Bio-Techne, Germany), and the uppermost value (1000 pg TNF-α/mL) was set to 100 %. As an additional PC, the TNF-α-forming capacity of NR8383 cells was confirmed by stimulation with lipopolysaccharide (LPS; 0.1 μg/mL, Sigma Aldrich, Germany). Finally, the direct effects of the test materials on L929 cells were assessed using 50 µL of the supernatants from cell-free NM-containing wells.

### H_2_O_2_ formation

H_2_O_2_ synthesized by NR8383 cells and released into the supernatant was quantified in the Amplex red assay by measuring the formation of resorufin. All chemicals used within this assay were supplied by Sigma Aldrich (Germany). NR8383 cells were seeded in 96-well microtitre plates at a density of 3 × 10^5^ cells/well and incubated under standard cell culture conditions. After 24 h, the medium was replaced by 100 μL of the test materials suspended in KRPG buffer. Immediately after incubation with the test material suspensions, 100 μL/well of a freshly prepared reaction mix containing 0.1 mM Ampliflu, 2 mM NaN_3_, and 2 U/mL horseradish peroxidase was added and incubated at 37 °C for 90 min. KRPG buffer was used as NC and 180 μg/mL zymosan as PC. Accuracy of the reactions was controlled with a 30 μM H_2_O_2_ standard concentration prepared from a 30 % stock solution freshly prepared from a 30 % H_2_O_2_ stock solution. OD was measured at 570 nm and 620 nm (reference value), corrected for background absorbance of cell free-particle controls and converted into absolute concentrations of H_2_O_2_ using the molar extinction coefficient of resorufin (54,000 L × mol^−1^ × cm^−1^).

### Statistical analysis

In vitro data were generated in triplicates and at least three independent repetitions were carried out. Data were expressed as mean ± standard deviation (SD) and analysed with Graph Pad Prism software (Version 6; GraphPad Software Inc., USA). To test for significance, test values were compared to those from non-treated vehicle controls using 2-way ANOVA and Dunnett’s post hoc multiple comparison test. Test results with p ≤ 0.05 were assessed as significant (*). Additionally, the significance of results was assessed by comparison to the effects elicited by equal particle concentrations of the negative benchmark material corundum. Finally, the concordance of the in vitro NR8383 AM assay results and the outcome of in vivo inhalation studies was assessed using Cooper statistics [93].

### Prediction model

A prediction model was developed to enable using the data from the in vitro NR8383 AM assay to distinguish active from passive NMs.

#### Test material categorization as ‘active’ or ‘passive’ using STIS data

The primary (‘gold standard’) distinction between active and passive (nano)materials was based upon in vivo rat STIS data that were available for the test materials [10, 11, 79–82]. Only for AlOOH, the available in vivo inhalation data were not recorded after 5-day inhalation exposure to rats (i.e., following the STIS test protocol), but in a 28-day sub-acute inhalation study conducted in accordance with OECD TG 412 [58].

NOAECs < 10 mg/m^3^ that were assigned on account of any adverse treatment-related inflammatory reaction elicited in the rat upon 5-day inhalation exposure of the given NM under the testing conditions of the well-defined STIS resulted in test material assignment as active. This definition considered any inflammatory effect recorded by haematology, BALF evaluation, and/or lung histopathology. Test materials were assigned as passive if no treatment-related findings were recorded at 10 mg/m^3^ or even higher concentratons in the STIS. Accordingly, their NOAEC was 10 mg/m^3^ or higher (indicated as ‘≥’ in Table 3). Since AlOOH was tested for 28 days (i.e., for four consecutive 5-day exposure periods), the NOAEC that Pauluhn [58] recorded for this material (i.e., 3 mg/m^3^) was converted to a 5-day NOAEC by multiplying it by a factor of four (i.e., 12 mg/m^3^) [94].

The recorded STIS NOAECs and lowest-observed-adverse-effect concentrations (LOAECs) for those materials that elicited inflammatory effects in at least one test group are provided in Table 3. Based thereupon, quartz DQ12, TiO_2_ NM-105, ZnO NM-111, all CeO_2_ test materials, as well as the three SiO_2_ NMs without surface functionalization (i.e., SiO_2_.naked, SiO_2_ NM-200 and NM-203) were categorized as in vivo active, and all other test materials were categorized as in vivo passive (*cf.* “In vitro studies and test material assignment as active or passive” section for further details on the available in vivo data). The prediction model for the in vitro NR8383 AM assay was conceived to provide the best possible correlation to this in vivo categorization.

#### Determination of in vitro lowest-observed effect concentration

The data obtained for all four in vitro endpoints (or ‘parameters’, i.e., LDH, GLU, TNF-α, H_2_O_2_) were recorded separately for all test materials. As described in “Statistical analysis” section, the significance of individual test results was assessed by comparing them both to the results obtained for the non-treated cell controls (Table 3) and the negative benchmark material corundum (Additional file 1: Table S1). The lowest dose at which significant effects were recorded for a given endpoint-specific test result was termed in vitro LOAEC.

#### Definition of in vitro threshold value

Consistent with the ‘vector model’ described by Rehn et al. [54, 55], in which the in vitro dosages were set to cover the mass-based in vivo concentration range that is likely to be taken up by rat AMs (*cf.* “Background” section), a mass-based dose metric was primarily used both for the in vitro testing and to assess the in vivo STIS findings. However, the effects of NMs on AMs may not be directly related to the particle mass. While the dose metric of particle volume appears relevant to determine AM overloading [23, 24], at test material concentrations below the threshold for volume overload, the effects of NMs on AMs appear to be mainly conveyed by the particle surface. Accordingly, surface area has been suggested as a more appropriate dose metric for the in vitro testing of NMs [95–99].

To convert the mass-based test material concentrations into surface area-based concentrations, the applied mass concentrations (μg/mL) were multiplied with the respective test material’s surface area (m^2^/g) as assessed by the method of Brunauer Teller and Emmett (BET). The BET surface area is an approximation of the actual surface area of primary particles and/or agglomerates to the biologically accessible surface [100]. This conversion resulted in the dose metric of particle surface area per volume (mm^2^/mL).

For the in vitro distinction between active and passive test materials, a threshold value of 4000 μm^2^ per NR8383 cell (mean diameter: 12.5 μm) was set at the cellular level (see also “Test protocol, prediction model, and threshold values” section for explanation). This value corresponds to 1200 mm^2^ particle surface area per 3 × 10^5^ cells which actively gather particles at the bottom of a well of a 96-well culture plate (equivalent to 3600 mm^2^/cm^2^ cell culture surface area). As the cell culture wells are filled with 200 μL culture medium the above threshold value amounts to 6000 mm^2^/mL and this dimension was used to convert applied test material concentrations (expressed in μg/mL) into the in vitro LOAEC values as shown in Table 3. Any significant in vitro LOAEC recorded below this threshold value was interpreted as biologically relevant, i.e., NM-specific cellular effect. By contrast, a significant in vitro LOAEC that was only recorded at a test material concentration exceeding the threshold was interpreted as unspecific and being caused by cellular overload, but not by particle-specific toxicity.

#### In vitro definition of ‘active’ and ‘passive’ test materials

To strengthen the robustness of the assay and to rule out incidental or borderline reactions by founding assignments on one affected endpoint alone, NMs were only assigned as active if significant in vitro LOAECs below the threshold value of 6000 mm^2^/mL were recorded for at least two of the four in vitro endpoints (e.g., elevated LDH and TNF-α). The occurrence of no or only one significant in vitro LOAEC below the threshold value of 6000 mm^2^/mL resulted in NM assignment as passive. (Nevertheless, the occurrence of one significant finding alone may be noteworthy for the further investigation of specific mechanisms of toxicity.)

## Results

### Test material characterization

Table 1 presents the test materials’ primary particle size (PPS; determined by transmission electron microscopy (TEM) or scanning electron microscopy (SEM)), BET surface area, and their particle size distribution (mean or modal values, d_50_ and d_90_) in water, KRPG buffer or F-12K medium. In general, particle sizes measured with tracking analysis in H_2_O were larger than the corresponding PPS. This was assessed as being either an indication for agglomeration and/or the material-dependent detection limit of the tracking analysis method.

**Table 1:** Primary characterisation of the test materials and agglomeration in biological fluids

Test materials	Size	Surface area	Form	Size in H_2_O	Size in KRPG	Size in F-12 K	Conc. in F-12K
	SEM, TEM [nm]	BET [m^2^/g]		[nm]	[nm]	[nm]	[Particles/mL]
TiO_2_ NM-105	28	47	P	d_50_ 167 d_90_ 246	Not detectable	Not detectable	Not detectable
ZnO NM-111	82	15	P	Not detectable	Not detectable	Not detectable	Not detectable
Nano-CeO_2_	40	33	P	d_50_ 159 d_90_ 263	Not detectable	Not detectable	Not detectable
Al-doped CeO_2_	2–160	46	P	d_50_ 132 d_90_ 189	Not detectable	Not detectable	Not detectable
CeO_2_ NM-211	10–20	66	P	d_50_ 130 d_90_ 199	Not detectable	Not detectable	Not detectable
CeO_2_ NM-212	10–20	27	P	d_50_ 136 d_90_ 195	Not detectable	Not detectable	Not detectable
SiO_2_.naked	5–50	200	S	d_50_ 48 d_90_ 91	d_50_ 58 d_90_ 99	d_50_ 59 d_90_ 94	2.3 × 10^11^
SiO_2_.PEG	8–45	200	S	d_50_ 54 d_90_ 83	Not detectable	Not detectable	Not detectable
SiO_2_.amino	5–50	200	S	d_50_ 50 d_90_ 75	d_50_ 90 d_90_ 148	d_50_ 72 d_90_ 120	2.9 × 10^11^
SiO_2_.phosphate	5–50	200	S	d_50_ 52 d_90_ 83	d_50_ 67 d_90_ 107	d_50_ 68 d_90_ 106	1.0 × 10^11^
SiO_2_ NM-200	10–20	189	P	d_50_ 129 d_90_ 177	Not detectable	Not detectable	Not detectable
SiO_2_ NM-203	5–30	200	P	d_50_ 151 d_90_ 210	d_50_ 174 d_90_ 305	Not detectable	Not detectable
AlOOH	40	47	P	d_50_ 163 d_90_ 243	Not detectable	Not detectable	Not detectable
BaSO_4_ NM-220	25	41	P	d_50_ 86 d_90_ 130	Not detectable	Not detectable	Not detectable
Fe_2_O_3_ (hematite)	15	98	P	d_50_ 88 d_90_ 159	d_50_ 64 d_90_ 142	d_50_ 113 d_90_ 328	2.7 × 10^8^
ZrO_2_.TODA	3–15	117	S	d_50_ 77 d_90_ 113	Not detectable	Not detectable	Not detectable
ZrO_2_.acrylate	9	117	S	d_50_ 172 d_90_ 244	Not detectable	Not detectable	Not detectable
DPP orange N	30–400 × 10–50	64	P	d_50_ 108 d_90_ 179	d_50_ 129 d_90_ 183	d_50_ 89 d_90_ 230	1.3 × 10^8^
Pigment blue 15:1	17	53	P	d_50_ 191 d_90_ 293	d_50_ 193 d_90_ 290	d_50_ 173 d_90_ 263	2.1 × 10^9^
Graphite nanoplatelets	<30 μm (flakes)	74	P	Not detectable	Not detectable	Not detectable	Not detectable

Primary particle size and BET surface area values were taken from Landsiedel et al. [11], Kroll et al. [17], Singh et al. [87, 88], and Rasmussen et al. [89, 90]

Form of the as-supplied test materials: Powder (P); Suspension (S)

Size measurements of the NM preparationss were carried out with NanoSight tracking analysis in double destilled water, in KRPG buffer, and F-12K culture medium; not detectable: not detectable due to low or missing particle concentration

Conc.: Concentration of nanoparticles as indicated by the NanoSight software; values were multiplied by the dilution factor

For most test materials, the numbers of particles dispersed in KRPG buffer or F-12K medium after 16-h incubation underscored the detection limit for reliable tracking analysis (indicated as ‘not detectable’ in Table 1). Only for SiO_2_.naked, SiO_2_.amino, SiO_2_.phosphate, SiO_2_ NM-203, the inorganic pigment Fe_2_O_3_, and the two organic pigments DPP Orange N and Pigment Blue 15:1, measurable numbers of dispersed particles were recorded in KRPG buffer or F-12K medium at the end of the incubation period (Table 1):

Despite their small size and low light scattering properties, colloidal SiO_2_.naked and its negatively charged surface functionalized variants SiO_2_.amino and SiO_2_.phosphate were observable with tracking analysis in H_2_O, and particle sizes matched the upper PPS. Under testing conditions, i.e., in KRPG buffer and in F-12K medium, particle sizes of these materials only slightly increased, gravitational settling was minimal, and only few agglomerates became visible at the bottom of the culture dish. Particle concentrations in F-12K medium ranged from 1.0 × 10^11^ particles/mL for SiO_2_.phosphate to 2.9 × 10^11^ particles/mL for SiO_2_.amino. By contrast, the neutral SiO_2_.PEG completely agglomerated in all media and no dispersed nanoparticles were detectable in either KRPG buffer or F-12K medium.

Unlike the colloidal SiO_2_ NMs, the dry-powder amorphous SiO_2_, i.e., precipitated SiO_2_ NM-200 and pyrogenic SiO_2_ NM-203, required extensive ultrasonication to destroy large agglomerates. Nevertheless, dispersed SiO_2_ NM-200 or NM-203 nanoparticles were not detectable by tracking analysis in F-12K medium at the end of the incubation period (but in KRPG buffer for SiO_2_ NM-203). For both dry-powder SiO_2_ test items, gravitational settling occurred that was more pronounced than for the colloidal SiO_2_ NMs.

Within 16 h, low, but detectable amounts of dispersed particles of inorganic Fe_2_O_3_ or organic DPP Orange N were observed in the F-12K supernatant with mean particle sizes of 113 and 89 nm, respectively. By comparison, the PPSs were 15 nm for Fe_2_O_3_ and 30–400 × 10–50 nm for DPP Orange N. Even though slight colourations remained in the medium after agglomerate sedimentation, this could be corrected for using cell-free controls and, therefore, did not affect the OD measurements. Particle concentrations in F-12K medium were 2.7 × 10^8^ for Fe_2_O_3_ and 1.3 × 10^8^ for DPP Orange N, i.e., by a factor of 10^3^ lower than the concentrations recorded for the dispersed colloidal SiO_2_ NMs.

Pigment Blue 15:1 could be dispersed in H_2_O by ultrasonication (180 μg/mL resulted in 5 × 10^10^ particles/mL; d_50_:191 nm as compared to a PPS of 17 nm). However, less than 5 % of that particle number concentration were observed in F-12K medium after 16 h incubation (180 μg/mL resulted in 2.1 × 10^9^ particles/mL; d_50_: 173 nm) in spite of the addition of 0.05 % (w/v) bovine serum albumin during ultrasonication. The vast majority of this nanosized pigment formed blue agglomerates.

### Cellular uptake of the test materials

**Fig. 1:**
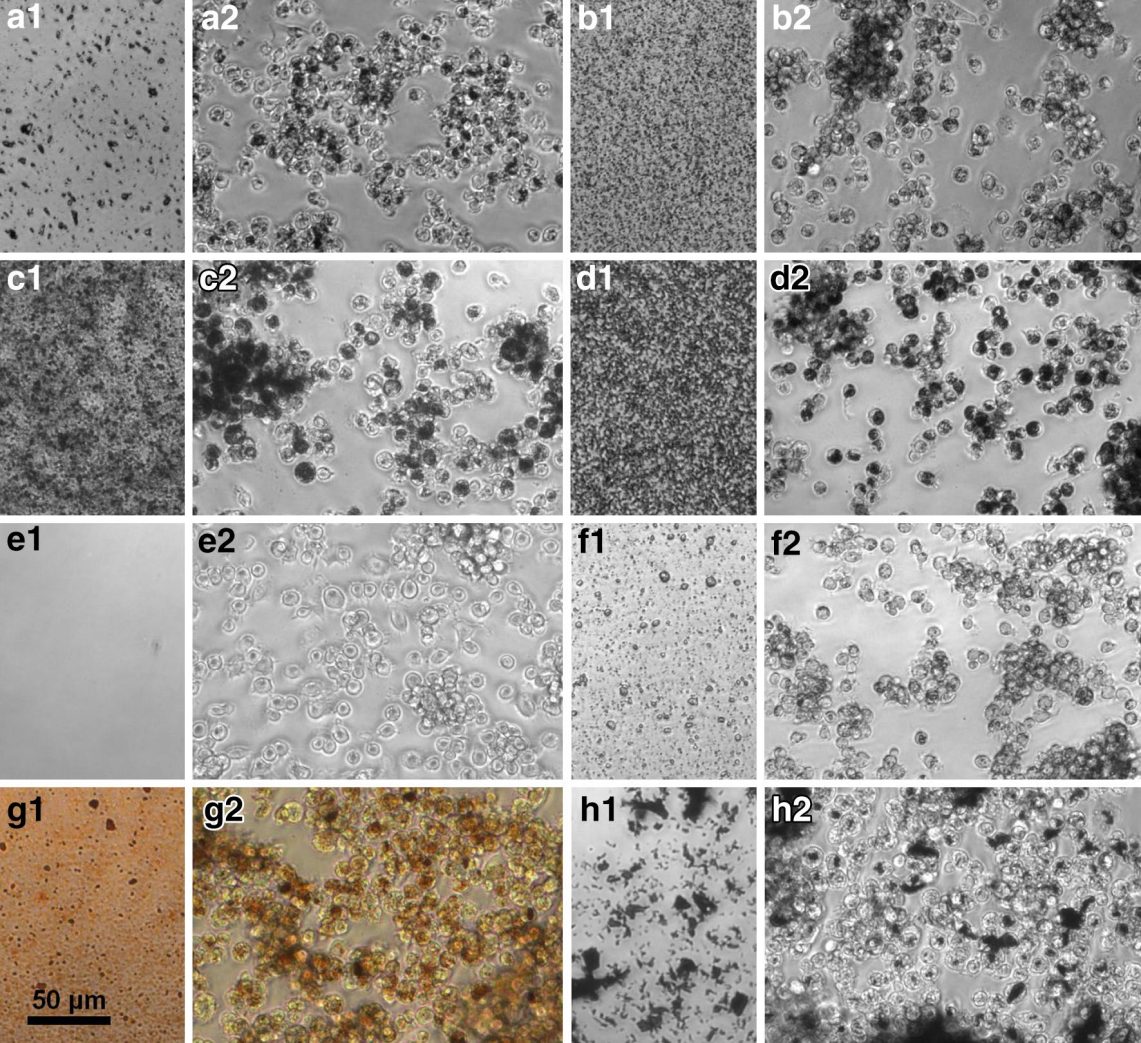
Test material sedimentation and uptake by NR8383 rat alveolar macrophages. A Corundum; B Quartz DQ12; C TiO_2_ NM-105; D CeO_2_ NM-212; E SiO_2_.naked; F BaSO_4_ NM-220; G DPP Orange N; H Graphite nanoplatelets. Phase contrast micrographs show settled particles at the bottom of cell-free wells in 96-well plates (**a1**–**h1** on the *left side* of the individual images) and corresponding particle-laden NR8383 cells at the end of the 16-h incubation period with the same particle concentration **a2**–**h2** on the *right side* of the individual images). Note that the uptake of particles (loaded with 90 μg/mL) appears complete as viewed by light microscopy, except for SiO_2_.naked (loaded with 22.5 μg/mL), where settled particles are hardly visible

Agglomerated particles settled to the bottom of the culture vessel where they were visible with phase contrast optics. As a rule, the test materials agglomerated and settled gravitationally within the 16-h incubation period. This settling occurred within minutes for corundum, within less than 1 h for quartz DQ12, 3–4 h for ZrO_2_.TODA, 6 h for ZrO_2_.acrylate, or by the end of the 16 h incubation period for TiO_2_ NM-105 and all four CeO_2_ NMs. Further as a rule, up to the highest test material concentration of 180 μg/mL, all test materials were completely engulfed by the NR8383 AMs (that are present at the bottom of the cell culture vessels) by the end of the 16-h incubation period (*cf.* Fig. 1 for phase-contrast images of settled particles and test material-laden NR8383 AMs for corundum, quartz DQ12, TiO_2_ NM-105, CeO_2_ NM-212, SiO_2_.naked, BaSO_4_ NM-220, DPP Orange N, and graphite nanoplatelets).

As exceptions to this rule, additional and specific observations were made for the following materials. Whereas complete cellular uptake was recorded for quartz DQ12 up to test concentrations of 90 μg/mL, many particles were visible outside deteriorated cells at 180 μg/mL underlining the pronounced cytotoxic effect of this material. AlOOH formed branched agglomerates under culture conditions which settled as a fluffy layer. For the different types of CeO_2_ NMs, cellular uptake was complete up to a concentration of 90 μg/mL, whereas very few agglomerates remained between the NR8383 cells at 180 μg/mL. Graphite nanoplatelets have a strong light absorbance. Nevertheless this carbonaceous NM could be measured colourimetrically up to a concentration of 180 μg/mL since the substance precipitated. Although largely ingested by NR8383 macrophages, some large flocs of graphite nanoplatelets were not fully internalised, but were contacted by surrounding cells.

In conclusion, for most test materials, complete cellular uptake was recorded by the end of the incubation period at all tested concentrations. Only for SiO_2_.naked, SiO_2_.amino and SiO_2_.phosphate, relevant proportions had not sedimented within 16 h (and hence could not be taken up by the AMs). Further, for graphene nanoplatelets, that did sediment, the highest test substance concentration was not fully internalized by the cells.

### In vitro studies and test material assignment as active or passive

In the following, for each test material, the in vitro data are presented and evaluated to assign the material as either passive or active, and this in vitro assignment is directly compared to the outcome of the corresponding available in vivo studies and the resulting in vivo categorization as active or passive test materials.

Table 2 provides an overview of the data collected in the in vitro NR8383 AM assay determining extracellular release of LDH, GLU, TNF-α, and H_2_O_2_ listing all endpoint-specific data recorded at all test material concentrations (expressed in mass per volume metrics; i.e. µg/mL). In further processing these data, Table 3 presents the endpoint-specific significant in vitro LOAECs recorded for each test material, expressing these values both in mass per volume metrics and in relation to the BET surface area (i.e., mm^2^/mL). Thereby, Table 3 reveals whether significant effects occurred below the threshold value of 6000 mm^2^/mL, and if so, how many parameters were significantly affected below the threshold for a given test material. Based thereupon, Table 3 further provides the in vitro NM assignments as either active or passive with contrasting juxtaposition to the STIS NOAECs, LOAECs and in vivo categorizations as active or passive.

**Table 2:** Effects of the negative control corundum, the positive control quartz DQ12, and the 20 test materials on the NR8383 cells

Test material	μg/mL	LDH	GLU	TNF-α	ROS/H_2_O_2_
		[% of PC]	[% of PC]	[% standard]^a^	[% of PC]
Corundum	0	18.5 ± 2.7	3.7 ± 0.6	0.0 ± 0.0	2.7 ± 1.7
	22.5	17.3 ± 3.6	3.7 ± 0.6	14.3 ± 4.8	1.0 ± 0.3
	45	20.2 ± 3.1	4.2 ± 0.8	14.7 ± 5.7	1.4 ± 0.8
	90	23.2 ± 2.5	4.5 ± 0.2	16.5 ± 7.1	1.9 ± 0.9
	180	25.8 ± 2.5	4.7 ± 0.5	26.0 ± 5.1	2.2 ± 1.3
Quartz DQ12	0	20.5 ± 1.3	3.7 ± 0.6	0.0 ± 0.0	2.7 ± 1.7
	22.5	18.5 ± 2.6	4.2 ± 1.7	8.3 ± 5.1	3.6 ± 2.7
	45	31.3 ± 6.0	6.4 ± 1.5	47.6 ± 12.1*	3.4 ± 2.6
	90	66.4 ± 6.3*	15.0 ± 3.4*	80.9 ± 10.4*	4.6 ± 2.3
	180	95.4 ± 5.2*	31.7 ± 4.9*	95.8 ± 1.4*	5.7 ± 3.8
TiO_2_ NM-105	0	18.0 ± 2.2	4.3 ± 3.5	0.0 ± 0.0	4.9 ± 4.3
	22.5	14.0 ± 2.9	3.5 ± 0.9	19.2 ± 17.2	4.9 ± 4.3
	45	26.2 ± 6.1	5.4 ± 1.7	25.8 ± 16.9	5.4 ± 4.9
	90	53.6 ± 5.9*	11.5 ± 1.7*	55.4 ± 7.6*	5.0 ± 3.1
	180	69.2 ± 1.1*	18.6 ± 0.7*	59.6 ± 6.6*	4.6 ± 4.8
ZnO NM-111^b^	0	27.4 ± 1.4	4.2 ± 2.5	0.0 ± 0.0	0.3 ± 0.2
	2.8/22.5	34.4 ± 1.4	4.1 ± 2.0	18.5 ± 5.0	0.0 ± 0.1
	5.6/45	36.7 ± 1.1*	7.7 ± 0.9	19.1 ± 0.5	0.3 ± 0.5
	11.3/90	39.1 ± 1.7*	18.2 ± 8.6*	13.2 ± 2.1	0.4 ± 0.6
	22.5/180	127.0 ± 2.7*	19.9 ± 4.6*	89.1 ± 1.4*	0.3 ± 0.7
nano-CeO_2_	0	25.0 ± 2.9	5.2 ± 0.4	0.0 ± 0.0	1.3 ± 0.0
	22.5	18.5 ± 5.1	4.2 ± 0.2	20.5 ± 9.8	1.3 ± 0.2
	45	28.4 ± 3.3	4.5 ± 0.1	35.8 ± 23.9*	1.2 ± 0.5
	90	45.1 ± 2.1*	5.4 ± 0.2	89.3 ± 10.6*	2.8 ± 1.0
	180	74.3 ± 9.3*	9.8 ± 0.5*	96.2 ± 5.3*	4.7 ± 1.1
Al-doped CeO_2_	0	25.0 ± 2.9	5.2 ± 0.4	0.0 ± 0.0	1.3 ± 0.0
	22.5	26.0 ± 8.4	5.0 ± 0.1	64.9 ± 35.2*	3.1 ± 0.1
	45	56.5 ± 11.2*	8.6 ± 0.8	84.7 ± 14.1*	3.2 ± 0.5
	90	84.1 ± 15.0*	19.8 ± 1.3*	89.8 ± 9.9*	4.2 ± 1.5
	180	91.1 ± 14.7*	25.0 ± 0.3*	88.4 ± 12.6*	5.7 ± 0.5
CeO_2_ NM-211	0	25.0 ± 2.9	5.2 ± 0.4	0.0 ± 0.0	1.3 ± 0.0
	22.5	26.5 ± 8.5	6.0 ± 0.1	70.8 ± 11.1*	0.6 ± 0.9
	45	38.9 ± 7.8	5.7 ± 0.2	65.4 ± 13.3*	0.3 ± 0.7
	90	56.5 ± 7.0*	6.7 ± 0.2	75.0 ± 17.2*	2.7 ± 1.2
	180	75.0 ± 7.1*	9.9 ± 0.1*	85.6 ± 15.6*	4.4 ± 1.7
CeO_2_ NM-212	0	25.0 ± 2.9	5.2 ± 0.4	0.0 ± 0.0	1.3 ± 0.0
	22.5	17.9 ± 7.8	4.9 ± 0.4	63.8 ± 29.4*	1.8 ± 0.8
	45	29.2 ± 12.0	6.0 ± 2.2	66.7 ± 26.3*	1.1 ± 2.1
	90	45.7 ± 14.6*	5.4 ± 0.2	76.9 ± 22.0*	2.9 ± 0.2
	180	69.0 ± 16.6*	9.9 ± 0.8*	88.5 ± 10.7*	1.8 ± 0.5
SiO_2_.naked	0	19.9 ± 1.2	2.7 ± 2.0	0.0 ± 0.0	3.7 ± 2.1
	22.5	40.5 ± 2.6*	5.0 ± 1.2	79.6 ± 3.0*	4.6 ± 2.9
	45	87.6 ± 9.8*	14.7 ± 3.7*	93.4 ± 5.7*	10.7 ± 3.8*
	90	100.1 ± 5.8*	24.6 ± 4.5*	77.3 ± 30.7*	13.9 ± 1.6*
	180	83.8 ± 19.0*	25.8 ± 3.7*	65.0 ± 33.2*	14.1 ± 2.3*
SiO_2_.PEG	0	19.9 ± 1.2	2.7 ± 2.0	0.0 ± 0.0	3.7 ± 2.1
	22.5	22.8 ± 5.2	3.1 ± 0.6	26.2 ± 0.9	1.5 ± 4.3
	45	30.5 ± 7.0	3.5 ± 1.1	26.2 ± 2.4	6.7 ± 4.1
	90	69.9 ± 7.9*	10.8 ± 2.4*	61.6 ± 6.4*	10.2 ± 5.7
	180	87.8 ± 7.8*	24.4 ± 4.5*	91.2 ± 4.4*	14.6 ± 6.8*
SiO_2_.amino	0	19.9 ± 1.2	2.7 ± 2.0	0.0 ± 0.0	3.7 ± 2.1
	22.5	22.0 ± 1.8	3.0 ± 0.5	44.4 ± 3.8*	4.4 ± 1.8
	45	70.4 ± 2.3*	8.2 ± 1.5*	99.1 ± 1.1*	5.5 ± 3.0
	90	96.2 ± 13.1*	17.9 ± 5.4*	99.1 ± 1.1*	6.6 ± 3.5
	180	99.7 ± 12.2*	25.8 ± 8.4*	98.3 ± 0.8*	13.0 ± 6.8*
SiO_2_.phosphate	0	19.9 ± 1.2	2.7 ± 2.0	0.0 ± 0.0	3.7 ± 2.1
	22.5	16.6 ± 2.5	2.3 ± 0.5	25.0 ± 4.9	10.1 ± 4.2
	45	29.8 ± 12.5	3.1 ± 0.3	19.9 ± 5.1	25.4 ± 6.2*
	90	41.7 ± 21.7*	4.8 ± 1.4	53.0 ± 24.7*	37.2 ± 8.1*
	180	48.6 ± 22.1*	7.3 ± 5.0*	78.7 ± 8.8*	36.8 ± 6.4*
SiO_2_ NM-200	0	13.2 ± 1.2	4.8 ± 3.1	0.0 ± 0.0	0.4 ± 0.2
	22.5	61.0 ± 2.1*	14.0 ± 5.6*	34.2 ± 25.0*	0.5 ± 1.5
	45	90.8 ± 18.7*	26.6 ± 9.1*	46.2 ± 21.4*	0.5 ± 1.0
	90	94.9 ± 13.8*	30.0 ± 4.8*	61.3 ± 22.6*	0.4 ± 1.0
	180	88.2 ± 15.0*	23.5 ± 7.2*	72.0 ± 18.6*	0.3 ± 1.1
SiO_2_ NM-203	0	13.2 ± 1.2	4.8 ± 3.1	0.0 ± 0.0	0.4 ± 0.2
	22.5	83.9 ± 23.6	33.7 ± 6.4*	50.2 ± 16.7	0.7 ± 1.1
	45	88.1 ± 13.8	34.3 ± 5.1*	49.8 ± 14.2	0.7 ± 1.2
	90	85.3 ± 16.3*	30.1 ± 8.7*	50.0 ± 18.3	0.5 ± 0.8
	180	86.7 ± 16.2*	32.2 ± 5.7*	64.4 ± 18.0*	0.3 ± 0.7
AlOOH	0	20.5 ± 1.5	3.6 ± 0.1	0.0 ± 0.0	0.3 ± 0.1
	22.5	20.7 ± 1.6	3.5 ± 0.1	5.2 ± 0.3	0.0 ± 0.0
	45	22.7 ± 1.6	3.3 ± 0.1	13.4 ± 1.8	0.0 ± 0.0
	90	39.9 ± 0.1*	4.4 ± 0.1	14.8 ± 0.2	0.2 ± 0.3
	180	61.5 ± 0.4*	6.6 ± 0.2	37.6 ± 4.1*	0.2 ± 0.2
BaSO_4_ NM-220	0	22.2 ± 0.9	3.0 ± 0.3	0.0 ± 0.0	1.8 ± 2.0
	22.5	24.0 ± 3.0	4.0 ± 0.3	0.0 ± 0.0	1.8 ± 2.1
	45	27.5 ± 3.3	3.6 ± 0.4	0.0 ± 0.0	1.8 ± 2.4
	90	30.2 ± 4.6	3.8 ± 0.3	0.0 ± 0.0	1.7 ± 2.0
	180	37.1 ± 5.9	3.8 ± 0.4	2.5 ± 0.1	1.7 ± 1.8
Fe_2_O_3_ (hematite)	0	22.1 ± 1.9	1.2 ± 1.0	0.0 ± 0.0	2.0 ± 0.8
	22.5	20.0 ± 0.6	1.6 ± 0.7	17.5 ± 4.2	3.1 ± 0.6
	45	25.4 ± 5.4	2.1 ± 0.5	16.5 ± 0.4	3.5 ± 0.4
	90	26.0 ± 0.6	2.2 ± 0.4	32.1 ± 10.3*	4.1 ± 0.6
	180	25.6 ± 0.9	2.8 ± 0.5	64.3 ± 33.2*	4.0 ± 1.0
ZrO_2_.TODA	0	22.7 ± 3.5	2.8 ± 2.1	0.0 ± 0.0	1.1 ± 0.6
	22.5	25.7 ± 3.5	2.9 ± 1.1	21.8 ± 3.9	1.8 ± 0.5
	45	39.9 ± 7.9*	3.0 ± 1.1	21.8 ± 6.4	5.8 ± 1.6
	90	70.2 ± 8.4*	5.4 ± 0.8	68.7 ± 15.5*	8.3 ± 1.4*
	180	81.2 ± 12.8*	7.2 ± 0.2	87.9 ± 10.5*	8.0 ± 2.3*
ZrO_2_.acrylate^c^	0	22.7 ± 3.5	2.8 ± 2.1	0.0 ± 0.0	1.1 ± 0.6
	35	29.6 ± 8.4	3.3 ± 1.2	25.3 ± 4.7	0.7 ± 2.1
	70.5	49.0 ± 13.6*	4.9 ± 1.6	48.5 ± 5.5*	7.4 ± 0.5
	141	73.9 ± 11.3*	11.7 ± 3.7*	90.5 ± 1.7*	15.2 ± 2.8*
	283	80.8 ± 4.1*	19.7 ± 5.8*	90.8 ± 6.2*	22.5 ± 4.7*
DPP orange N	0	22.1 ± 1.9	1.2 ± 1.0	0.0 ± 0.0	2.0 ± 0.8
	22.5	19.9 ± 1.9	1.8 ± 0.6	25.9 ± 16.5	1.0 ± 0.1
	45	22.7 ± 1.9	2.3 ± 0.6	48.7 ± 37.7*	1.5 ± 0.4
	90	25.7 ± 1.1	3.2 ± 0.4	66.0 ± 57.0*	2.2 ± 0.4
	180	28.4 ± 0.5	5.3 ± 0.7	81.5 ± 30.9*	3.3 ± 1.3
Pigment blue 15:1	0	20.5 ± 1.3	4.2 ± 0.2	Not determined	0.2 ± 0.2
	22.5	17.4 ± 3.1	6.0 ± 0.3		0.0 ± 0.0
	45	20.0 ± 3.6	8.4 ± 3.5		0.0 ± 0.0
	90	41.0 ± 7.2*	10.6 ± 0.6*		0.0 ± 0.0
	180	88.0 ± 7.9*	21.9 ± 2.2*		0.0 ± 0.0
Graphite nanoplatelets	0	22.1 ± 1.9	1.2 ± 1.0	0.0 ± 0.0	2.0 ± 0.8
	22.5	24.2 ± 2.6	2.6 ± 1.0	2.2 ± 2.9	1.4 ± 0.8
	45	26.3 ± 1.9	3.3 ± 1.2*	8.4 ± 4.8	2.1 ± 1.2
	90	29.9 ± 0.4	5.7 ± 1.9*	30.2 ± 17.2*	4.1 ± 2.0
	180	37.8 ± 3.3	11.8 ± 2.5*	74.8 ± 9.4*	7.1 ± 1.3

To test for significance, test values were compared to those from non-treated vehicle controls using 2-way ANOVA with Dunnett’s post hoc multiple comparison test, and p ≤ 0.05 was assessed as significant (*)

^a^Measurements of TNF-α are expressed as L929 fibroblast lysis relative to non-treated medium controls, which were set to 0 %

^b^Due to its high bioactivity, ZnO NM-111 was tested for release of LDH and TNF-α at 2.8–22.5 μg/mL, whereas GLU and H_2_O_2_ formation were assessed at 22.5–180 μg/mL

^c^For technical reasons, ZrO_2_.acrylate was assessed at 35–283 μg/mL

**Table 3:** Comparison of significant in vitro LOAECs to NOAECs and LOAECs recorded in rat short-term inhalation studies

Test materials			In vitro NR8383 AM assay									STIS		
Class	Name	BET [m^2^/g]	LOAEC [µg/mL]				LOAEC [mm^2^/mL]					NOAEC [mg/m^3^]	LOAEC [mg/m^3^]^a^	Ref^d^
			LDH	GLU	TNF-α	ROS H_2_O_2_	LDH	GLU	TNF-α	ROS H_2_O_2_	Threshold <6000	Threshold <10		
Micron-sized crystalline silica	Quartz DQ12	8	90	90	45	n.s.	*720*	*720*	*360*	n.s.	***3***	***0.1***	***1.0***	[106]
Active metal oxide NMs	TiO_2_ NM-105	47	90	90	90	n.s.	*4230*	*4230*	*4230*	n.s.	***3***	***<2***	***2.0***	[10]
	ZnO NM-111	15	5.6	90	22.5	n.s.	*84*	*1350*	*338*	n.s.	***3***	***0.5***	***2.5***	[11]
	nano-CeO_2_	33	90	180	45	n.s.	*2970*	n.s.	*1485*	n.s.	***2***	***0.5***	***2.5***	[11]
	Al-doped CeO_2_	46	45	90	22.5	n.s.	*2070*	*4140*	*1035*	n.s.	***3***	***0.5***	***2.0***	[11]
	CeO_2_ NM-211	66	90	180	22.5	n.s.	*5940*	n.s.	*1485*	n.s.	***2***	***<0.5***	***5.0***	[111]
	CeO_2_ NM-212	27	90	180	22.5	n.s.	*2430*	n.s.	*608*	n.s.	***2***	***<0.5***	***5.0***	[111]
Amorphous SiO_2_ NMs	SiO_2_.naked	200	22.5	45	22.5	45	*4500*	9000	*4500*	9000	***2***	***2.5***	***10***	[11]
	SiO_2_.PEG	200	90	90	90	180	18000	18000	18000	36000	0	≥50	n.r.	[11]
	SiO_2_.amino	200	45	45	22.5	180	9000	9000	*4500*	36000	1	≥50	n.r.	[11]
	SiO_2_.phosphate	200	90	180	90	45	18000	n.s.	18000	9000	0	≥50	n.r.	[11]
	SiO_2_ NM-200	189	22.5	22.5	22.5	n.s.	*4253*	*4253*	*4253*	n.s.	***3***	***1***	***5***	[80]
	SiO_2_ NM-203	200	22.5	22.5	22.5	n.s.	*4500*	*4500*	*4500*	n.s.	***3***	***1***	***5***	[80]
Passive metal oxide and metal sulphate NMs	AlOOH	105	90	n.s.	180	n.s.	9450	n.s.	18900	n.s.	0	(3)^b^	(28)^b^	[58]
	BaSO_4_	41	n.s.	n.s.	n.s.	n.s.	n.s.	n.s.	n.s.	n.s.	0	≥50	n.r.	[11]
	Fe_2_O_3_ (hematite)	98	n.s.	n.s.	90	n.s.	n.s.	n.s.	8266	n.s.	0	≥30	n.r.	[79]
	ZrO_2_.TODA	117	45	n.s.	90	90	*5265*	n.s.	10530	10530	1	≥50	n.r.	[11]
	ZrO_2_.acrylate	117	70.5	141	70.5	141	8249	16497	8249	16497	0	≥50	n.r.	[11]
Nanosized organic pigments	DPP Orange N	64	n.s.	n.s.	45	n.s.	n.s.	n.s.	*2880*	n.s.	1	≥30	n.r.	[79]
	Pigment Blue 15:1	53	90	90	n.d.^c^	n.s.	*4770*	*4770*	n.d.^c^	n.s.	***2***	≥30	n.r.	[79]
Carbonaceous NM	Graphite nanoplatelets	74	n.s	45	90	n.s	n.s	*3330*	6660	n.s	1	≥10	n.r.	[82]

For all parameters, the significant in vitro LOAECs (significance as compared to the vehicle controls) are shown, both in mass/volume (µg/mL) and surface area/volume (mm^2^/mL) dose metrics (n.s.: no signficance). The surface area/volume-based values were calculated by multiplying the mass/volume values by the respective NM’s BET surface area. Surface area/volume-based values that undercut the in vitro threshold of 6000 mm^2^/mL are provided in italics. For test material assignment as either active (significant LOAEC < 6000 mm^2^/mL, ≥ 2 of 4 parameters affected) or passive (0 or only 1 parameter affected), the frequency of affected parameters is indicated in the column ‘threshold < 6000 mm^2^/mL’. Further, available rat STIS NOAEC values are provided for all test materials (and LOAEC values, if effects were observed). For both the in vitro and in vivo data, values indicating ‘activity’ are highlighted in bold italic

^a^n.r.: If no effects were observed in the STIS up to the highest tested concentration, no LOAEC was recorded (n.r.)

^b^Since AlOOH was tested for 28 days (i.e., four consecutive 5-day exposure periods), the NOAEC that Pauluhn [58] recorded for this material (i.e., 3 mg/m^3^) was converted to a 5-day NOAEC by multiplying it by a factor of four [94]. Accordingly, the calculated 5-day NOAEC of 12 mg/m^3^ indicates passivity

^c^For technical reasons, TNF-α was not determined (n.d.) for Pigment Blue 15:1. However, since significant in vitro LOAECs below the threshold were recorded for LDH and GLU, this does not impair its assignment as ‘in vitro active’

^d^Numbers in brackets apply to (short-term) inhalation studies as listed in the reference section

For a better overview, Tables 2 and 3, just as the following subsections of “In vitro studies and test material assignment as active or passive”, are subdivided into the following sections: First, the data for the NC and PC are provided, i.e., corundum (Al_2_O_3_) and quartz DQ12. Next, the data for the seven metal oxide NMs that were identified as active in vitro are presented, i.e., TiO_2_ NM-105, ZnO NM-111, and all four tested CeO_2_ NMs. This is followed by the data recorded for the amorphous SiO_2_ NMs. The subsequent section presents the data for the four metal oxide and metal sulphate NMs that were identified as passive in vitro, i.e., AlOOH, BaSO_4_ NM-220, Fe_2_O_3_, and both surface-functionalized ZrO_2_. The final two sections present the data recorded for the two nanosized organic pigments and graphite nanoplatelets.

#### Corundum

##### In vitro, corundum is assigned as passive

Corundum induced very slight dose-dependent increases of LDH, TNF-α and H_2_O_2_, all of which were not significantly different from the non-treated controls. This lack of effects confirms the suitability of corundum as NC and as a negative benchmark material.

##### In vivo categorization confirms corundum passivity

Previous rat STISs underlined the inert nature of respirable corundum particles (aerosol concentration 20 mg/m^3^; 2-week exposure, 5 days/week; 5 h/day) [77]. Also in rat instillation studies, corundum proved to be a chemically inert particle which hardly elicited any pulmonary inflammatory or fibrogenic effects [101, 102]. However, high intratracheal instillation doses of 7.5 mg/rat lung [102] or 5 mg/100 g body weight [103], which are both in the overload range, elicited BALF changes that mainly consisted of elevated polymorphonuclear neutrophil (PMN) counts. In line with these findings, the United States National Institute for Occupational Safety and Health (NIOSH) has set an occupational exposure limit (OEL) of 5 mg/m^3^ for respirable corundum (summarized by Krewski et al. [104]). Taken together, corundum is categorized as passive in vivo, which confirms in vitro passivity.

#### Quartz DQ12

##### In vitro, quartz DQ12 is assigned as active

The in vitro macrophage toxicity of quartz DQ12 is well-known [54, 105]. Quartz DQ12 induced dose-dependent releases of LDH, GLU, and TNF-α, significant well below the threshold value of 6000 mm^2^/mL. Even though quartz DQ12 hardly elicited any extracellular H_2_O_2_ formation, the findings confirm its suitability as PC and results in quartz DQ12 assignment as active material.

##### In vivo categorization confirms quartz DQ12 activity

In a rat 28-day sub-acute inhalation study, a NOAEC of 0.1 mg/m^3^ was recorded for alpha-quartz (median particle size: 1.7 µm) [106]. Due to its progressive inflammatory, fibrogenic and genotoxic effects, micron-sized quartz DQ12 is widely used as a PC for in vivo studies [102, 107]. As PC, it was tested at one (high) concentration, each, in two rat 5-day STISs. At 25 mg/m^3^, quartz DQ12 produced progressively severe effects over the 3-month post-exposure period [80] with similar findings recorded at 100 mg/m^3^ [81]. Generally, the results observed in the STIS at the end of the post-exposure observation periods resembled those recorded in sub-chronic inhalation studies [108]: Macrophage, monocyte, PMN, and also lymphocyte counts were increased in the lung parenchyma and BALF, which coincided with elevated levels of total protein and enzyme activities [LDH, alkaline phosphatase (AP), γ-glutamyltransferase (GGT) and N-acetyl-glucosaminidase (NAG)]. Upon intratracheal instillation in rats, pulmonary fibrosis was observed even after bolus doses of only 0.15–0.3 mg [102, 109]. Such dosages may be reached under STIS conditions by aerosol concentrations that are far lower than 10 mg/m^3^. In conclusion, quartz DQ12 is categorized as active material, which confirms the in vitro assignment.

#### Active metal oxide NMs (TiO_2_, ZnO, CeO_2_)

##### In vitro, TiO_2_ NM-105 is assigned as active

TiO_2_ NM-105 elicited dose dependent increases of LDH, GLU, and TNF-α (significant in vitro LOAECs at 4230 mm^2^/mL, each), whereas H_2_O_2_ formation did not differ from the vehicle control. Since significant in vitro LOAECs below the threshold value of 6000 mm^2^/mL were recorded for 3 of the 4 parameters, TiO_2_ NM-105 is assigned as active NM.

##### In vivo categorization confirms TiO_2_ NM-105 activity

In a number of different STISs assessing both coated and uncoated TiO_2_ NMs, pulmonary inflammatory changes were recorded, with a NOAEC for TiO_2_ NM-105 of <2 mg/m^3^ and a LOAEC of 2 mg/m^3^. Most prominent findings were BALF increases in total cell count, PMNs, and AMs. Additionally, total protein and the activities of LDH, ALP, GGT, NAG were increased [10, 11, 81]. Based upon these in vivo STIS data, TiO_2_ NM-105 is categorized as active NM, which confirms the in vitro assignment.

##### ZnO NM-111 is assigned as in vitro active

ZnO NM-111, that is coated with triethoxycaprylylsilane, is a NM with a comparably small BET surface area of 15.1 m^2^/g. This material dissolves in acidic environments thereby shedding zinc ions [79]. Maximum cytotoxicity (release of LDH) was already observed at 22.5 μg/mL (significant in vitro LOAEC at 5.6 μg/mL, i.e., 84 mm^2^/mL), while the parameter GLU became significant at 90 μg/mL (1350 mm^2^/mL) and TNF-α induction was at its maximum between 22.5 and 45 μg/mL (in vitro LOAEC 338 mm^2^/mL). TNF-α formation was inhibited at higher concentrations due to progressive cell degradation. Extracellular H_2_O_2_ formation induced by ZnO NM-111 was in the same range as the concurrent vehicle control value. In parallel studies, the non-coated core material ZnO NM-110 was even more toxic than coated ZnO NM-111 for all parameters tested (data not shown). Since significant in vitro LOAECs below the threshold value of 6000 mm^2^/mL were recorded for 3 of the 4 parameters, ZnO NM-111 (just as ZnO NM-110) is assigned as active NM.

##### In vivo categorization confirms ZnO NM-111 activity

Coated ZnO NM-111 elicited extensive signs of inflammation in the BALF upon 5-day inhalation exposure to 2.5 mg/m^3^ (NOAEC: 0.5 mg/m^3^). The most prominent findings were increased total cell counts caused by invaded PMNs, lymphocytes, and monocytes [11]. Total protein concentration and enzyme activities (GGT, LDH, ALP and NAG) were increased as well, just as a number of inflammatory mediators, i.e., cytokine-induced neutrophil chemoattractant 1 (CINC-1, the rat homologue to IL-8), clusterin, cystatin C, granulocyte chemotactic protein 2 (GCP-2), and MCP-1. In a 14-day STIS, 8 mg/m^3^ uncoated ZnO NM-110 (only this concentration tested) also elicited pulmonary inflammatory effects so that a 14-day NOAEC < 8 mg/m^3^ was assigned to ZnO NM-110 [110]. Based upon these in vivo STIS data, ZnO NM-111 (just as ZnO NM-110) is categorized as active NM, which confirms the in vitro assignment.

##### All four CeO_2_ NMs (Al-doped CeO_2_, nano-CeO_2_, CeO_2_ NM-211, and CeO_2_ NM-212) are assigned as in vitro active

All four types of CeO_2_ NMs dose-dependently increased LDH and GLU release. The in vitro LOAECs recorded for LDH were significant and below the threshold value of 6000 mm^2^/mL for all four CeO_2_ NMs. Additionally, for Al-doped CeO_2_, the in vitro LOAEC recorded for GLU was significant and below the threshold value. Evaluation of these two parameters that indicate cell membrane damage resulted in the following ranking of cytotoxicity (when using the particle surface area-based LOAECs): Al-doped CeO_2_ > CeO_2_ NM-212 > nano-CeO_2_ > CeO_2_ NM-211. H_2_O_2_ formation was far less pronounced (and never significant), but also headed by Al-doped CeO_2_. Finally, the in vitro LOAECs recorded for TNF-α induction were significant and below the 6000 mm^2^/mL threshold value for all four CeO_2_ NMs, albeit with a slightly different ranking, i.e., CeO_2_ NM-212 > Al-doped CeO_2_ > CeO_2_ NM-211 = nano-CeO_2_. Accordingly, the findings indicate different biological activities and potencies of in vitro cellular effects of the four tested CeO_2_ NMs with Al-doped CeO_2_ and CeO_2_ NM-212 eliciting more pronounced effects than nano-CeO_2_ or CeO_2_ NM-211. Since significant in vitro LOAECs below the threshold value of 6000 mm^2^/mL were consistently recorded for the two parameters LDH and TNF-α (and additionally for GLU in the case of Al-doped CeO_2_), all four CeO_2_ NMs are assigned as active.

##### In vivo categorization confirms activity for all four CeO_2_ NMs

All four CeO_2_ NMs induced a pronounced transient inflammation at 0.5 mg/m^3^, and effects increased at higher doses up to 25 mg/m^3^ [11, 111]. Three days post-exposure, relative PMN fractions in the BALF increased to 76 and 79 % for Al-doped CeO_2_ and nano-CeO_2_, respectively, and concomitantly, the BALF total protein levels were elevated [11]. For all four CeO_2_ NMs, a NOAEC of <0.5 mg/m^3^ was assigned [11, 111]. Accordingly, all four CeO_2_ NMs are categorized as active, which confirms the in vitro assignment.

For an in-depth in vitro-in vivo comparison of the test results recorded for the four CeO_2_ NMs, the in vitro data were further compared to specific BALF findings that are directly related to AM-induced alterations of the in vivo rat lung, i.e. absolute values of total cells, AM, PMN as well as total protein concentration. By contrast to the above-mentioned particle surface area-based in vitro ranking, the following ranking of cytotoxicity is achieved when using the mass-based in vitro data: Al-doped CeO_2_ > CeO_2_ NM-211 = nano-CeO_2_ > CeO_2_ NM-212. As presented in further detail in the Additional file 1: Table S2, the BALF findings [11, 111] allow the following conclusions on in vivo AM-related pulmonary alterations: Al-doped CeO_2_ and nano-CeO_2_ dose dependently decreased the number of AMs in the BALF and increased PMN counts and total protein concentration alike, whereas the corresponding effects elicited by CeO_2_ NM-211 and CeO_2_ NM-212 were approx. 60 % lower over the entire concentration range. Hence, even though the four different CeO_2_ NMs were assigned the same STIS NOAEC of <0.5 mg/m^3^, there were gradual differences in BALF parameters at nominally identical conditions of NM in the inspired air. Also the lung burdens recorded for the four different CeO_2_ NMs upon 5-day inhalation exposure differed [11, 111]. These differences are partly reflected by the in vitro tests in which Al-doped CeO_2_ was clearly more bioactive than e.g., CeO_2_ NM-212, at least in the low to middle dose range.

#### Amorphous SiO_2_ NMs

##### Colloidal SiO_2_.naked is assigned as in vitro active and its surface-functionalized variants (SiO_2_.PEG, SiO_2_.amino, and SiO_2_.phosphate) as in vitro passive

For all four colloidal SiO_*2*_ NMs, significant in vitro LOAECs were recorded for all four test parameters with the exception of GLU for SiO_2_.phosphate. However, as a rule, these significant LOAECs by far exceeded the threshold value of 6000 mm^2^/mL. Only for SiO_2_.naked, LDH and TNF-α attained 4500 mm^2^/mL, each, and for SiO_2_.amino, TNF-α attained this same value. Accordingly, only for the non-surface functionalized SiO_2_.naked, two parameters with significant in vitro LOAECs below 6000 mm^2^/mL, i.e., the threshold for biologically relevant, particle-specific (i.e., non cellular overload-induced) effects, were recorded. In conclusion, SiO_2_.naked is assigned as in vitro active, whereas its surface-functionalized variants SiO_2_.PEG, SiO_2_.amino, and SiO_2_.phosphate are assigned as passive.

##### In vivo categorizations confirm both SiO_2_.naked activity and the passivity of the surface-functionalized SiO_2_

At 10 and 50 mg/m^3^, SiO_2_.naked evoked dose-dependent signs of inflammation in the rat STIS [11]. Three days after the final exposure, the most predominant significant effect in the BALF was an increased PMN count that was accompanied by slightly elevated BALF lymphocyte counts and moderately increased numbers of blood PMNs. In histopathological evaluation, multifocal macrophage aggregates were observed in the lung that exacerbated towards a slight multi-focal pulmonary inflammation by the end of the 3-week exposure free period. Accordingly, a NOAEC of 2.5 mg/m^3^ was assigned to SiO_2_.naked [11]. By contrast, no adverse effects were observed after inhalation exposure to up to 50 mg/m^3^ SiO_2_.PEG, SiO_2_.phosphate, or SiO_2_.amino, and the NOAEC for these materials was assessed as being ≥50 mg/m^3^ [11]. Based upon the in vivo STIS data, SiO_2_.naked is categorized as active NM and SiO_2_.PEG, SiO_2_.phosphate, and SiO_2_.amino as passive NMs, which confirms the in vitro assignment.

##### SiO_2_ NM-200 and NM-203 are assigned as in vitro active

Both precipitated SiO_2_ NM-200 and pyrogenic SiO_2_ NM-203 consistently elicited significant LDH, GLU and TNF-α release at 22.5 μg/mL, each. For all three parameters, this corresponds to significant in vitro LOAECs of 4253 and 4500 mm^2^/mL, for SiO_2_ NM-200 and NM-203, respectively. Accordingly, for both dry-powder amorphous SiO_2_ NMs, two parameters, each, ranged below the threshold value of 6000 mm^2^/mL, and both SiO_2_ NM-200 and NM-203 are assigned as active.

##### In vivo categorization confirms SiO_2_ NM-200 and NM-203 activity

For precipitated SiO_2_ NM-200 and pyrogenic SiO_2_ NM-203, STIS data were available for precipitated Zeosil^®^ 45 and pyrogenic Cab-O-Sil^®^ M5. The equivalence of these materials to SiO_2_ NM-200 and NM-203, respectively, has been established based upon concordance in production process and minimum degree of material purity as well as comparability of specific surface area and agglomerate size [80, 112, 113]. In the rat STIS published by Arts et al. [80], test material concentrations of 1, 5, and 25 mg/m^3^ were applied, and 1 mg/m^3^ was recorded as NOAEC for both SiO_2_ NM-200 and NM-203, whereas 5 mg/m^3^ was assessed as LOAEC. For SiO_2_ NM-200, increased weights of the lungs and lung-associated lymph nodes (LALNs) as well as an inflammatory response of the lung tissue were recorded at 5 and 25 mg/m^3^ that were accompanied by dose-dependently increased PMN counts, enzyme activities, and protein levels in the BALF. For SiO_2_ NM-203, increased lung weights and hypertrophy of the bronchiolar epithelium were significant in the 5 and 25 mg/m^3^ test groups. The LALNs contained increased silica levels, and, again, dose-dependently increased PMN and macrophage counts in the BALF indicated inflammatory reactions. For both materials, all effects were fully reversible within 3 months post-exposure [80]. Based upon the in vivo STIS data, SiO_2_ NM-200 and NM-203 are categorized as active, which confirms the in vitro assignment.

#### Passive metal oxide and metal sulphate NMs (AlOOH, BaSO_4_, Fe_2_O_3_, ZrO_2_)

##### AlOOH is assigned as in vitro passive

AlOOH (boehmite) elicited dose-dependent and significant increases of LDH and TNF-α (significant in vitro LOAECs: 9450 and 18,900 mm^2^/mL, respectively). Hence, even though significant LOAECs were recorded for two parameters, both values ranged well above the threshold value of 6000 mm^2^/mL indicating that the observed effects were elicited under in vitro cellular overload conditions. Accordingly, these effects are assessed as not-particle specific, and AlOOH is assigned as passive.

##### In vivo categorization confirms AlOOH passivity

The AlOOH NM included in the present study (PPS: 40 nm; BET surface area: 105 m^2^/g) and a smaller AlOOH variant (PPS: 10 nm; BET surface area: 182 m^2^/g) were submitted to a 28-day rat sub-acute inhalation study followed by a 3-month post-exposure observation period [58]. In this study, no adverse effects were recorded at AlOOH aerosol concentrations of 0.4 or 3 mg/m^3^. Pulmonary inflammation (recorded by significantly altered BALF parameters, increased lung and LALN weights and histopathological findings) was observed at 28 mg/m^3^ AlOOH. However, these effects were only elicited by cumulative doses exceeding approx. 1 mg AlOOH/g lung at the end of the 28-day exposure period [58]. Since the concentration dependence and time-course changes of aluminum lung burden demonstrated a precipitous increase in elimination half-time at aerosol concentrations of 28 mg/m^3^, Pauluhn assessed these findings as being consistent with pulmonary overload [58]. An earlier intratracheal instillation study confirmed these results indicating a NOAEC of 0.6 mg/rat lung, whereas reversible increases in BALF PMN and total protein levels were recorded at bolus doses of 1.2 mg/rat lung [84]. Since AlOOH was tested for 28 days (i.e., four consecutive 5-day exposure periods), the NOAEC of 3 mg/m^3^ that Pauluhn [58] recorded for this material was converted to a 5-day NOAEC by multiplying it by a factor of four [94]. In accordance with this calculated 5-day NOAEC of 12 mg/m^3^, AlOOH is categorized as passive, which confirms the in vitro assignment.

##### BaSO_4_ NM-220 is assigned as in vitro passive

BaSO_4_ NM-220 did not elicit any cellular effects that differed from the vehicle or corundum controls. Therefore, BaSO_4_ NM-220 is assigned as passive.

##### In vivo categorization confirms BaSO_4_ NM-220 passivity

No adverse effects were observed in a rat STIS after inhalation exposure to up to 50 mg/m^3^ BaSO_4_ NM-220 [11]. Accordingly, BaSO_4_ NM-220 is categorized as passive, which confirms the in vitro assignment.

##### Fe_2_O_3_ is assigned as in vitro passive

For Fe_2_O_3_ (hematite), the only significant LOAEC was recorded for TNF-α (8266 mm^2^/mL). This one in vitro LOAEC further exceeded the threshold value of 6000 mm^2^/mL. Of note, the cytotoxic effects elicited by this nanosized Fe_2_O_3_ were equal to or lower than those of its bulk counterpart, whereas the amount of sedimented test material appeared comparable (data not shown). Since only one parameter was affected, nanosized Fe_2_O_3_ is assigned as passive.

##### In vivo categorization confirms Fe_2_O_3_ passivity

Inhalation exposure to up to 30 mg/m^3^ Fe_2_O_3_ (or its non-nanosized counterpart; data not shown) in a STIS did not cause any adverse effects in the rat lung as was determined by BALF evaluation, hematology and histopathological evaluation [79]. Based upon this in vivo study, Fe_2_O_3_ (just as its non-nanosized counterpart) is categorized as passive, which confirms the in vitro assignment.

##### ZrO_2_.TODA and ZrO_2_.acrylate are assigned as in vitro passive

ZrO_2_.TODA and ZrO_2_.acrylate dose-dependently and significantly increased LDH, TNF-α, and H_2_O_2_ formation, and ZrO_2_.acrylate additionally GLU. However, apart from the LDH value recorded for ZrO_2_.TODA (in vitro LOAEC: 5265 mm^2^/mL), which laid just below the 6000 mm^2^/mL threshold, all other significant in vitro LOAECs recorded for either ZrO_2_.TODA or ZrO_2_.acrylate exceeded the threshold value. Accordingly, both ZrO_2_.TODA and ZrO_2_.acrylate are assigned as passive.

##### In vivo categorization confirms ZrO_2_.TODA and ZrO_2_.acrylate passivity

No adverse effects were observed in a rat STIS after inhalation exposure to up to 50 mg/m^3^ ZrO_2_.acrylate or ZrO_2_.TODA [11]. In rat intratracheal instillation studies, bolus dose-NOAECs of 0.6 and 1.2 mg/rat lung were recorded for ZrO_2_.TODA and ZrO_2_.acrylate, respectively [114]. Based upon these in vivo studies, both ZrO_2_.TODA and ZrO_2_.acrylate are categorized as passive, which confirms the in vitro assignment.

#### Nanosized organic pigments

##### DPP Orange N is assigned as in vitro passive

DPP Orange N elicited a significant increase of TNF-α (in vitro LOAEC 2880 mm^2^/mL) that ranged well below the threshold value of 6000 mm^2^/mL. However, since LDH, GLU, and H_2_O_2_ formation were not significantly altered, the premise that at least two of the four parameters had to be altered to indicate NM activity was not met. Of note, also the bulk counterpart to DPP Orange N, DPP Orange B, did not elicit relevant cytotoxicity in the in vitro NR8383 AM assay (data not shown). Accordingly, DPP Orange N (and DPP Orange B) are assigned as passive.

##### In vivo categorization confirms DPP Orange N passivity

Inhalation exposure to up to 30 mg/m^3^ DPP Orange N in a STIS did not cause any adverse effects in the rat lung as was determined by BALF evaluation, hematology and histopathological evaluation [79]. For the respective bulk material DPP Orange B, high aerosol concentrations of 30 mg/m^3^ slightly increased total cell count, PMN, MCP-1 and osteopontin in the BALF (data not shown). Based upon these in vivo studies, DPP Orange N (just as DPP Orange B) are categorized as passive, which confirms the in vitro assignment.

##### Pigment Blue 15:1 is assigned as in vitro active

Pigment Blue 15:1 elicited dose-dependent increases of LDH and GLU with significant LOAECs of 4770 mm^2^/mL, each. (The slightly blue colouration of the supernatants could be corrected for via cell-free controls.) Accordingly, two parameters had significant LOAECs ranging below the threshold value of 6000 mm^2^/mL. Based upon these in vitro findings, Pigment Blue 15:1 is classified as active.

##### In vivo categorization indicates Pigment Blue 15:1 passivity, thereby refuting the in vitro assignment as over-predictive

For Pigment Blue 15:1, a STIS NOAEC of 30 mg/m^3^ was recorded [79]. At this aerosol concentration, blue pigment-laden AMs were observed in the lung parenchyma and LALNs. Further, slight epithelial hypertrophy or hyperplasia was noted in terminal bronchioles that however were assessed as rather reflecting the challenged biological clearance mechanism than adverse reactions. All findings were fully reversible within the 3-week post-exposure period. Based upon this in vivo study, Pigment Blue 15:1 is categorized as passive. Accordingly, for this pigment, the in vitro NR8383 AM assay over-predicted its toxic potential in the rat STIS.

#### Graphite nanoplatelets

##### Graphite nanoplatelets are assigned as in vitro passive

Graphite nanoplatelets elicited significantly increased GLU and TNF-α levels (in vitro LOAECs, 3330 and 6660 mm^2^/mL, respectively. Accordingly, only one parameter (GLU) ranged below the threshold value of 6000 mm^2^/mL. Based upon these findings, graphite nanoplatelets are assigned as passive.

##### In vivo categorization confirms passivity of graphite nanoplatelets

Inhalation exposure to up to 50 mg/m^3^ graphite nanoplatelets in a STIS did not cause any adverse effects in the rat lung as was determined by BALF evaluation, hematology and histopathological evaluation [82]. Based upon this in vivo study, graphite nanoplatelets are categorized as passive, which confirms the in vitro assignment.

### Summary of in vitro–in vivo comparisons

In summary, for 19 of the 20 test materials, the in vitro NR838 AM assay addressing extracellular release of LDH, GLU, TNF-α and H_2_O_2_ correctly predicted in vivo activity or passivity in the rat STIS. Pigment Blue 15:1 was the only material that tested false positive: Based upon the significant in vitro LOAECs that ranged below the threshold value of 6000 mm^2^/mL for LDH and GLU (each: 4770 mm^2^/mL), 2 of the 4 in vitro parameters were positive. This resulted in Pigment Blue 15:1 assignment as active, whereas it had been categorized as passive based upon the high STIS NOAEC of 30 mg/m^3^.

By contrast, for SiO_2_.amino, ZrO_2_.TODA, DPP Orange N, and graphite nanoplatelets, only one parameter each tested positive with significant in vitro LOAECs ranging below the threshold value of 6000 mm^2^/mL. ZrO_2_.TODA only triggered LDH, graphite nanoplatelets only triggered GLU, and SiO_2_.amino and DPP Orange N each only triggered TNF-α release. By definition, this in vitro outcome resulted in their assignment as passive, and this result was confirmed by the in vivo data available for all four of these test materials.

Applying the Cooper statistics [93], the in vitro NR8383 AM assay performed under the conditions of the present study had a specificity of 91 % and a sensitivity of 100 % with an overall accuracy of 95 %. The rates for negative and positive prediction were 90 and 100 % (Table 4).

**Table 4:** Determination of the accuracy, sensitivity and specificity of the in vitro NR8383 alveolar macrophage assay

	Test material activity, STIS	Test material passivity, STIS	SUM	
Test material activity, in vitro	9	1	10	90 % positive prediction
Test material passivity, in vitro	0	10	10	100 % negative prediction
SUM	9	11	20	
	100 % sensitivity	91 % specificity		
	Accuracy 95 %			

Comparison of altogether 20 in vitro test results (*cf.* Table 3) to in vivo results from rat short-term inhalation studies applying the Cooper statistics [93]

Testing against a particulate benchmark material as a NC is mandatory in an empirical assay, since it provides information on the reliability and reproducibility of the behaviour of the cells under loading conditions. When evaluating all test results against the corresponding values recorded for the negative (albeit micron-sized) benchmark material corundum (Additional file 1: Table S1), Pigment Blue 15:1 was again assessed false positive. Since the test results obtained for corundum were slightly higher than those recorded for the particle-free vehicle control, the corundum-based evaluation resulted in some minor deviations from the vehicle control-based evaluation. This, however, resulted in a number of statistically relevant differences. Using the corundum-based evaluation, SiO_2_ NM-203 was assessed ‘false negative’ (only one significant LOAEC < 6000 mm^2^/mL was recorded, i.e., for GLU). Furthermore, ZrO_2_.TODA and SiO_2_.phosphate exhibited one positive finding, each. A sharp demarcation line between active and passive materials will be prone to erroneous decisions. Therefore, the premise had been set that at least two of the four parameters had to be altered to assign a material as active. Notwithstanding, false negative findings are detrimental for regulatory toxicity testing, and for this reason the statistical evaluation against untreated cells proved superior to the evaluation against the negative benchmark material corundum. Accordingly, in the subsequent discussion, only the vehicle-control-based evaluation is addressed.

## Discussion

In the present study, the in vitro NR8383 AM assay evaluating extracellular release of LDH, GLU, TNF-α and H_2_O_2_ under standardized conditions using protein-free culture medium proved highly accurate in distinguishing active from passive inorganic NMs or nanosized organic pigments. All nine test materials that had been categorized as active using the STIS NOAEC-based threshold value of <10 mg/m^3^ laid down by Arts et al. [33] were correctly identified as active. Further, of the 11 test materials that had been categorized as passive (i.e., inert) by a STIS NOAEC of 10 mg/m^3^ or higher, only one test material (Pigment Blue 15:1) was tested false positive in the in vitro NR8383 AM assay. For the other ten test materials that had been categorized as passive using the STIS NOAEC, the in vitro data correctly predicted in vivo passivity.

### Test protocol, prediction model, and threshold values

All 9 NMs that were assigned as active elicited elevated levels of extracellular LDH in the in vitro NR8383 AM assay. To increase the robustness of the assay and reduce the number of false positives, it had been defined that ≥2 of the 4 in vitro parameters had to be positive in order to assign a test material as active. For 3 of the 9 active NMs, each, the increased LDH levels coincided with increased GLU levels (SiO_2_ NM-200 and NM-203 and Pigment Blue 15:1), TNF-α levels (nano-CeO_2_ and CeO_2_ NM-211 and NM-212) or GLU plus TNF-α levels (TiO_2_ NM-105, ZnO NM-111, Al-doped CeO_2_). Also for the non-nanosized PC quartz DQ12, significantly elevated LDH, GLU and TNF-α were recorded, and a significant LDH level as sole affected parameter (that hence did not result in NM assignment as active) was recorded for ZrO_2_.TODA.

By contrast, significant H_2_O_2_ formation below the in vitro threshold value of 6000 mm^2^/mL was never recorded, neither as sole affected parameter, nor in combination with another affected parameter. Of note, however, a moderate to significant H_2_O_2_ formation was observed for the colloidal test materials (i.e., SiO_2_.naked and its surface functionalized variants as well as the surface functionalized ZrO_2_ NMs). The mechanism by which, e.g., SiO_2_.phosphate induces H_2_O_2_ formation remains unknown, but it may be related to non-specific receptor activation. However, apparently, it does not depend on the presence of diffusible nanoparticles, since only three of the H_2_O_2_-inducing NMs (i.e., SiO_2_.naked, SiO_2_.amino, and SiO_2_.phosphate) remained dispersed in the nanosize under the testing conditions of the present study.

It might be questioned whether the determination of ROS formation is essential for the in vitro NR8383 AM assay, especially since the STIS protocol does not foresee a directly corresponding parameter. Nevertheless, the authors of the present study suggest maintaining H_2_O_2_ formation as fourth parameter for the in vitro NR8383 AM assay. Different reaction patterns observed for different types of test materials may provide a first insight into their specific toxicological mechanisms which may further provide relevant information for the grouping of NMs [3, 33, 79] or the determination of adverse-outcome-pathways [115]. In this respect, also the observation that only some NMs elicited significant H_2_O_2_ formation requires further investigations.

AMs were selected as test system for the present study because the inflammatory effects that NMs may elicit in the rat lung upon short-term inhalation are considered to emanate at least in part from stimulated or compromised AMs. Accordingly, the mechanisms leading to pulmonary inflammation and which are reflected in the corresponding NOAECs are based upon AM effects. Importantly, in developing the test protocol for the in vitro NR8383 AM assay, high accuracy of the in vitro assay was achieved by expressing data using surface area-based dose metrics (mm^2^/mL) and by combining the in vitro threshold value of <6000 mm^2^/mL with the ‘at least 2 out of 4′ prediction model to assign a test material as active. The in vitro threshold value of 6000 mm^2^/mL has been set as a versatile measure to reflect the highest in vitro ‘non-overload’ dose under the conditions of the macrophage assay. Surface area-based dose metrics are widely accepted for the in vitro assessment of NMs since their cellular effects are conveyed by their surface [95–99, 116–120]. By contrast, prior to the present study, application of a surface area-based threshold value in an in vitro AM assay to distinguish active from passive NMs had not yet been suggested.

As outlined in “Definition of in vitro threshold value” section, the threshold of 6000 mm^2^/mL was derived from 4000 μm^2^/NR8383 cell. One must be aware that a sharply defined threshold value bears some uncertainty and should include some margin of safety. In this sense this threshold value has been derived from in vivo findings: Since NR8383 cells widely resemble in vivo rat AMs both in terms of morphology and biological reactivity, 4000 μm^2^/NR8383 cell may be multiplied with the total number of AMs per rat lung (i.e., 1–2 × 10^7^ [54]), resulting in a calculated particle surface area-based threshold for the rat lung of 4–8 × 10^10^ μm^2^ (corresponding to 0.04–0.08 m^2^). Since a rat lung weighs approx. 1 g [121], the same value applies when expressing the particle surface area-based threshold per gram lung tissue (i.e., 0.04–0.08 m^2^/g lung tissue). Interestingly, an earlier study addressing the sub-chronic pulmonary effects of inhaled TiO_2_ and BaSO_4_ NMs in rats suggested that the pulmonary overload of these poorly soluble particles begins at 0.02–0.03 m^2^/g lung tissue [95]. Hence, the in vitro threshold values applied in the present study is approx. twofold higher as it would be if the value from Tran et al. [95] was applied. This difference may be tolerable due to some uncertainties of the above-made calculations such as dynamically changing macrophage populations. Importantly, since materials that elicit adverse effects below the calculated threshold of 0.04–0.08 m^2^/g lung tissue are categorized as active in the present study, this higher threshold is conservative.

Taking into account the specific BET surface areas for different NMs, the calculated in vivo overload threshold of 0.04–0.08 m^2^/lung is equivalent to, e.g., 0.2–0.4 mg SiO_2_.naked (BET: 200 m^2^/g); 0.6–1.2 mg CeO_2_ NM-212 (BET: 66 m^2^/g); or 0.85–1.7 mg TiO_2_ NM-105 (BET: 47 m^2^/g). Accordingly, any in vivo adverse effect observed below these lung burdens should indicate test material-specific activity in the non-overload range.

However, to convert the in vivo overload threshold of 0.04–0.08 m^2^/lung to the mass-based aerosol concentration (i.e., mg/m^3^), NM lung deposition needs to be taken into account. Importantly, lung burden, as measured after 5-day exposure under STIS conditions, is not only concentration-dependent, but also material-dependent [11]. Hence, also the in vivo overload threshold will be material-dependent. For instance, if a specific NM is highly soluble or has a low pulmonary deposition, its apparent lung burden may be low, and the maximum ‘non-overload’ aerosol concentration may be higher than for an insoluble NM that further has a high pulmonary deposition. Moreover, material properties influencing lung clearance may play a role. For instance, exposure to 0.5 mg/m^3^ CeO_2_ NM-212 in a rat STIS resulted in a lung burden of 0.011 mg/lung directly after 5 days of exposure and decreased to 0.006 mg/lung by the end of the 21-day post-exposure period. By contrast, exposure to 5 and 25 mg/m^3^ of this same material yielded higher lung burdens with only little decrease during the post-exposure observation period, indicating impaired clearance [111]. Also the lung burdens recorded for CeO_2_ NM-212 were approx. twofold higher than the ones recorded for CeO_2_ NM-211 under identical conditions [111]. Accordingly, precise comparisons of in vitro and in vivo data recorded for a given test material require consideration of the measured lung burden (or of a reliable calculation thereof).

However, for practical reasons, i.e., for the prediction of NM activity or passivity under routine regulatory toxicity testing conditions, it appears advantageous to use the fixed in vitro threshold value of 6000 mm^2^/mL since it is easy to apply and further includes a margin of safety. Thereby, it is conservative: In the present study, it did not generate any false negative results. By contrast, for the time being, the occurrence of few false positive results (i.e., 1 out of 11 in the present study) cannot be avoided. Nevertheless, a low false positive rate does not prevent incorporation of the in vitro NR8383 AM assay into a regulatory framework for the hazard assessment of NMs.

Finally, the in vitro threshold of 6000 mm^2^/mL is compared to the threshold value of 10 μg/cm^2^ cell culture surface area that Kroll et al. [17] calculated as indicating in vitro cellular overload conditions. Of note, Kroll et al. related their threshold value to the entire rat lung surface area, but not to the pool of AMs. As presented above, the particle surface area-based in vitro threshold value of 6000 mm^2^/mL corresponds to 3600 mm^2^/cm^2^ cell culture surface area (for a cell culture volume of 200 μL). BET surface areas of the test materials applied in the present study ranged from 15 m^2^/g (i.e., 15 mm^2^/μg) for ZnO NM-111 to 200 m^2^/g (i.e., 200 mm^2^/μg) for the colloidal SiO_2_ NMs and pyrogenic SiO_2_ NM-203. Accordingly, for ZnO NM-111, the 6000 mm^2^/mL threshold value equals 240 μg/cm^2^; for test materials with a BET surface area of approx. 60 m^2^/g (CeO_2_ NM-211 or DPP Orange N) it equals 60 µg/cm^2^, and for the mentioned SiO_2_ NMs it equals 18 μg/cm^2^. Therefore, the in vitro threshold set in the present study is higher than the in vitro threshold set by Kroll et al. [17]. However, this is not surprising since the threshold set in the present study considers the pool of AMs as a cell compartment that actively collects particles, both in vitro and in vivo.

### Applicability domain of the in vitro NR8383 AM assay

All test materials that were identified as active in vitro or in vivo elicited inflammatory effects in the rat lung. In the STIS, this was indicated by elevated PMN counts in the BALF that frequently coincided with increased total protein values and enzyme activities. These early pro-inflammatory reactions became evident and were easily quantifiable by BALF analysis, whereas histopathological evaluation mostly revealed moderate alterations of the lung tissue that were possibly secondary to the inflammation. Accordingly, the in vitro determination of a NM’s activity allows predicting its inflammatory potential in the rat lung. This may include cytotoxic or AM-activating effects upon inhalation and deposition in the rat lung, and/or the evolvement of inflammatory responses secondary to its AM-activating effects.

Although the response of a NR8383 cell to a NM may, to a certain degree, be taken as being representative for other cells as well [68, 69], AM-independent, direct effects of NMs on epithelial cells or other pulmonary cells, which may also contribute to the overall pulmonary response to NMs (e.g., the epithelial cytokine response) are not covered by the in vitro NR8383 AM assay. Other in vitro test systems than AMs may reflect epithelial disorders evolving in the lung [122]. Nevertheless, also AMs may be suitable to assess such effects since they are either directly or indirectly involved in many pulmonary disorders. For instance, an impaired lung clearance resulting from compromised AM activity may lead to progressive pulmonary inflammation followed by e.g., fibrosis or neoplastic transformation. This is highlighted by the examples of quartz DQ12 or CeO_2_ NMs. These materials were assigned as active in the in vitro NR8383 AM assay. Upon chronic inhalation exposure, both materials may elicit fibrotic alterations of the lung. Of note, however, no neoplastic changes were reported upon treatment with CeO_2_ NMs [105, 109, 123].

As the spectrum of test materials selected for the present study and further in-house experience reveal, the in vitro NR8383 AM assay is applicable to poorly soluble low-toxicity particles (PSLT [124]) that are also called respirable granular biodurable particles (GBP [32]). Similarly, nuisance or inert dusts may be assessed, as long as they may be dispersed in the culture medium. Also particles retrieved from air filters, such as welding fumes or particles released from dental materials upon grinding, have been tested using the standard concentration range of 22.5–180 μg/mL. Particle agglomeration and gravitational settling were found to enhance the delivery of the applied dose to the AMs at the bottom of the culture vessels. In vitro particokinetics (i.e., particle size distribution and particle agglomeration) were controlled with phase contrast microscopy combined with tracking analysis of the supernatant. In fact, only 7 of the 20 test materials (SiO_2_.naked, SiO_2_.amino, SiO_2_.phosphate, SiO_2_ NM-203, Fe_2_O_3_, and the two organic pigments) remained at least partially dispersed in the culture media. As a result, the effective cellular dose of these materials cannot be determined with certainty using the simple microscopic characterization methods applied in the present study.

Nevertheless, there are indeed NMs which cannot be successfully tested in the in vitro NR8383 AM assay for technical reasons. For instance, while MWCNT NM-401 could be assessed (data not shown), other MWCNTs could not be dispersed sufficiently well to allow testing. Materials, which form agglomerates that float in suspension or which escape uptake by the AMs by adhering to the vessel walls or by gathering at the surface due to low buoyant density, can also not be assessed. Furthermore, materials which strongly interfere with the optical read out may cause problems, since the transmitted light levels may by too low to be corrected by the cell-free controls. Of note, however, the orange, red, and blue pigments could be evaluated since the AMs took them up and completely cleared the cell culture bottom from coloured agglomerates.

### Test material effects

In the following, the specific in vitro effects of the test materials observed in the in vitro NR8383 AM assay are compared to the findings reported for identical or comparable test materials available in the published literature.

#### Active metal oxide NMs (TiO_2_, ZnO, CeO_2_)

Generally, the in vitro findings recorded for the six metal oxide NMs that were identified as active (TiO_2_ NM-105, ZnO NM-111, and all four tested CeO_2_ NMs) stand in line with results from other in vitro or in vivo studies. Recently, anatase TiO_2_ NM-101 has been found to elicit weak activating effects on HL-60 cells which may represent human neutrophil-like cells [125]. In the same study, the ion-shedding ZnO NM-110 and Ag NM-300 elicited much more pronounced cytotoxicity. Consistent with these findings, also uncoated and coated ZnO NMs (*cf.* “Active metal oxide NMs (TiO2, ZnO, CeO2)” section) as well as Ag NM-300K (data not shown) were recorded as active in the in vitro NR8383 AM assay. Even though rat STIS data are unavailable for Ag NM-300K, these findings appear biologically relevant.

CeO_2_ NMs have a highly reactive surface which, apart from Ce^4+^ and Ce^3+^, is chiefly composed of oxygen and O^2−^. The change of oxidation state from 3^+^ to 4^+^ appears to determine the biological activity of CeO_2_ NMs. This may either result in ROS generating or ROS scavenging properties [21, 88]. Accordingly, different types of CeO_2_ NMs may be expected to elicit contradictory reactions, and different CeO_2_ NMs have indeed been observed to be either ROS/H_2_O_2_ protective [126, 127] or toxic in rats [123] or in different types of cultured cells [68].

However, the four different CeO_2_ NMs were consistently assigned as active both in vitro and in vivo. Nevertheless, based upon an in-depth evaluation of the data (*cf.* Additional file 1: Table S2), Al-doped CeO_2_ NMs elicited the most pronounced inflammatory reactions observed for CeO_2_ NMs in the present in vitro study (or in the published in vivo STIS data reflecting AM-based reactions in the rat lung). Especially Al-doping of CeO_2_ NMs has been recognized to strongly increase the O_2_-binding capacity of this material [128]. It may be assumed that O_2_-surface binding may be involved in its in vitro or in vivo toxicity. Further in vitro investigations should aim at addressing the specific mechanisms of toxicity or protective cellular effects that different types of CeO_2_ may elicit.

#### Amorphous SiO_2_ NMs

Of the four different colloidal SiO_2_ NMs submitted to the present study, only the unmodified SiO_2_.naked was tested positive in the rat STIS [11]. An instillation study with mice provided the same outcome for these same materials [129]. Furthermore, it revealed a weak effect on PMN recruitment into the lungs for SiO_2_.amino and SiO_2_.phosphate. In the present study, SiO_2_.amino elicited significant TNF-α release (only this one parameter affected), whereas for SiO_2_.phosphate none of the four parameters were affected. The effects of SiO_2_.naked recorded in the present study occurred in the absence of protein, since protein coating with fetal calf serum mitigates the toxicity of SiO_2_ NMs [21, 130]. It may be assumed that the protein-binding capacity of amorphous SiO_2_ may affect specific inflammatory or toxic responses in vivo. Concordantly, surface functionalization with PEG, amino or phosphate residue may reduce or affect protein binding, both in vitro and in vivo.

#### Passive metal oxide and metal sulphate NMs (AlOOH, BaSO_4_, Fe_2_O_3_, ZrO_2_)

Based upon the in vitro NR8383 AM data, AlOOH and BaSO_4_ NM-220 were assigned as passive. This result stands in full concordance with previous studies using a broad spectrum of different cell lines, including RAW264.7 macrophages, even though these studies were conducted in the presence of serum [17]. Also nanosized Fe_2_O_3_ (hematite) was assigned as passive. Its effects on LDH or GLU release were in the same range as its non-nanosized counterpart (data not shown). Hence, ‘nanosize’ of this inorganic pigment does not appear to augment its hazard potential.

In the present study, ZrO_2_.TODA, but not ZrO_2_.acrylate, elicited an increased LDH level (and a significant in vitro LOAEC was recorded for only this one parameter). ZrO_2_.TODA and ZrO_2_.acrylate also gradually differed with respect to the induced (albeit not significant) release of GLU and H_2_O_2_. This may point to differences caused by the different surface functionalizations. These minor findings do not correspond to the outcomes of the available STISs where neither material caused any effects up to 50 mg/m^3^ [11]. Nevertheless, different STIS lung burdens were measured for ZrO_2_.TODA (693 μg) and ZrO_2_.acrylate (169 μg) after 5-day inhalation exposure to identical aerosol concentrations of 50 mg/m^3^ [11], such that a final comparison cannot be made.

#### Nanosized organic pigments and graphite nanoplatelets

Coloured and dense materials are potentially difficult to evaluate in in vitro assays using colorimetric assays. Nevertheless, in the present study the two nanosized organic pigments (just as the inorganic red pigment Fe_2_O_3_) had mostly settled by the end of the incubation period, and further particles could be removed from the supernatant by centrifugation before the optical measurements. Thereby, an acceptable degree of variation was ensured. Also the influence of the few remaining particles could be circumvented by subtracting the values obtained for the cell-fee controls. Whereas the particles that remained in suspension (and hence did not sediment towards the cells) are not expected to affect the effective dose reaching the cells within the incubation period to a considerable extent, the precise effective cellular dose of these materials cannot be estimated.

For DPP Orange N only a dose-dependent formation of TNF-α was recorded, and just as its non-nanosized counterpart DPP Orange B, it was assigned as in vitro passive. Hence, also for this organic pigment there is no indication that the ‘nanosize’ increases its hazard potential.

Pigment Blue 15:1 significantly increased both LDH and GLU and the calculated values were below the threshold of 6000 mm^2^/mL. Thereby, this organic pigment delivered the only false positive result since it did not elicit adverse effects in vivo in the rat STIS up to aerosol concentrations of 30 mg/m^3^ [79]. Even though Pigment Blue 15:1 contains copper, this compound is tightly bound to the molecule, and ions are neither released in water, nor in biological media [79]. It must be underlined that the effects that Pigment Blue 15:1 elicited in vitro were not severe: Even a mean cellular load of 30 pg/AM (equivalent to 45 μg/mL) did not lead to cell membrane damage (*cf.* Table 2). However, at this concentration, the GLU release from the intact cells was already increased, and both parameters were significantly affected at 90 and 180 μg/mL. Even though no release of copper was measured, it is known that copper ions may affect GLU expression in cells [131–133]. Obviously, the disparity between the in vitro and STIS findings recorded for Pigment Blue 15:1 remains to be investigated in further detail.

## Conclusion

Investigating a broad spectrum of 18 inorganic NMs and 2 nanosized organic pigments, the in vitro NR8383 AM assay allowed distinguishing active from passive nanomaterials. AMs were selected as test system due to the predominant role these cells play in clearing the lung from inhaled particles. Further, many secondary pulmonary effects are also initiated or accompanied by AMs. The selected parameters, LDH, GLU, TNF-α and H_2_O_2_ formation and release, in combination with the ‘at least 2 out of 4’ prediction model proved easy to use and suitable for routine testing. Importantly, the model was highly efficient in predicting in vivo STIS hazard potential. Recently, application of the in vitro NR8383 AM assay within the DF4nanoGrouping Decision-making framework for the grouping and testing of NMs has shown that this assay also allows grouping NMs by biological activity [33, 79]. When integrated into a tiered testing approach, such as the DF4nanoGrouping, the in vitro NR8383 AM assay may substantially reduce the need for animal testing addressing the inhalation route of exposure. Further work should aim at validating this assay.

## Additional file

10.1186/s12951-016-0164-2 Comparison of significant in vitro LOAECs (significant as compared to the negative benchmark material corundum) to NOAECs and LOAECs recorded in rat STISs. **Table S2.** Bioactivity of four types of CeO_2_ NMs in rat STISs as compared to cellular effects recorded in the in vitro NR8383 AM assay.

## Abbreviations

AM: alveolar macrophage
AP: alkaline phosphatase
BALF: bronchoalveolar lavage fluid
BET: (method of) Brunauer, Teller, and Emmett
CINC: cytokine-induced neutrophil chemoattractant
DPP: diketopyrrololpyrrol
ECETOC: European Centre for the Ecotoxicology and Toxicology of Chemicals
ECHA: European Chemicals Agency
FCS: fetal calf serum
GBP: respirable granular biodurable (particles)
GGT: γ-Glutamyltransferase
GLU: β-Glucuronidase
IL: interleukin
JRC: Joint Research Centre (of the EU Commission)
KRPG: Krebs–Ringer phosphate glucose
LALN: lung-associated lymph node
LDH: lactate dehydrogenase
LOAEC: lowest-observed effect concentration
LPS: lipopolysaccharide
MCP: monocyte chemoattractant protein
MWCNT: multi-walled carbon nanotube
NAG: N-acetyl-glucosaminidase
NAPDH: nicotinamide adenine dinucleotide phosphate-oxidase
NC: negative control
NIOSH: National Institute for Occupational Safety and Health
NM: nanomaterial
OD: optical density
OECD: Organisation for Economic Co-operation and Development
OEL: occupational exposure limit
PBS: phosphate buffered saline
PC: positive control
PDGF: platelet-derived growth factor
PMN: polymorphonuclear neutrophil
PPS: primary particle size
PSLT: poorly soluble–low toxicity
REACH: registration, evaluation, authorisation and restriction of chemicals
ROS: reactive oxygen species
SD: standard deviation
STIS: short-term rat inhalation study
TG: test guideline
TGF-β: transforming growth factor β
TNF-α: tumour necrosis factor α
WPMN: working party on manufactured nanomaterials
