# Supplementary material for: An in vitro alveolar macrophage assay for predicting the short-term inhalation toxicity of nanomaterials
Source: J Nanobiotechnology. 2016 Mar 5;14:16. doi: 10.1186/s12951-016-0164-2 (PMC4779246; doi:10.1186/s12951-016-0164-2)
Supplement: Supplementary file 1 — 10.1186/s12951-016-0164-2 Comparison of significant in vitro LOAECs (significant as compared to the negative benchmark material corundum) to NOAECs and LOAECs recorded in rat STISs. Table S2. Bioactivity of four types of CeO2 NMs in rat STISs as compared to cellular effects recorded in the in vitro NR8383 AM assay. [file 12951_2016_164_MOESM1_ESM.doc]

**Supplementary Information to:**

**An *in vitro* alveolar macrophage assay for predicting the short-term inhalation toxicity of nanomaterials**

**Supplementary Information Table S1: Comparison of significant *in vitro* LOAECs (significant as compared to the negative benchmark material corundum) to NOAECs and LOAECs recorded in rat STISs**

| **Test materials** | | | ***In vitro* NR8383 AM assay** | | | | | | | | | **STIS** | | |
| --- | --- | --- | --- | --- | --- | --- | --- | --- | --- | --- | --- | --- | --- | --- |
| **Class** | **Name** | **BET** **[m^2^/g]** | **LOAEC [µg/mL]** | | | | **LOAEC [mm^2^/mL]** | | | | | **NOAEC [mg/m^3^]** | **LOAEC [mg/m^3^] ^a^** | **Ref. ^d^** |
|  |  |  | LDH | GLU | TNF-α | ROS H_2_O_2_ | LDH | GLU | TNF-α | ROS H_2_O_2_ | threshold <6000 | threshold <10 |  |  |
| Micron-sized crystalline silica | Quartz DQ12 | 8 | 90 | 90 | 45 | n.s. | **720** | **720** | **360** | n.s. | 3 | 0.1 | 1.0 | [106] |
| Active metal oxide NMs | TiO_2_ NM-105 | 47 | 90 | 90 | 90 | n.s. | **4230** | **4230** | **4230** | n.s. | 3 | <2 | 2.0 | [10] |
|  | ZnO NM-111 | 15 | 11.3 | 90 | 11.3 | n.s. | **169** | **1350** | **169** | n.s. | 3 | 0.5 | 2.5 | [11] |
|  | nano-CeO_2_ | 33 | 90 | n.s | 90 | n.s. | **2970** | n.s. | **2970** | n.s. | 2 | 0.5 | 2.5 | [11] |
|  | Al-doped CeO_2_ | 46 | 45 | 90 | 22.5 | n.s. | **2070** | **4140** | **1035** | n.s. | 3 | 0.5 | 2.0 | [11] |
|  | CeO_2_ NM-211 | 66 | 45 | 180 | 22.5 | n.s. | **2970** | 11880 | **1485** | n.s. | 2 | <0.5 | 5.0 | [111] |
|  | CeO_2_ NM-212 | 27 | 90 | n.s | 22.5 | n.s. | **2430** | n.s. | **608** | n.s. | 2 | <0.5 | 5.0 | [111] |
| Amorphous SiO_2_ NMs | SiO_2_.naked | 200 | 22.5 | 45 | 22.5 | 45 | **4500** | 9000 | **4500** | 9000 | 2 | 2.5 | 10 | [11] |
|  | SiO_2_.PEG | 200 | 90 | 180 | 90 | 90 | 18000 | 36000 | 18000 | 18000 | 0 | ≥50 | n.r. | [11] |
|  | SiO_2_.amino | 200 | 45 | 90 | 45.0 | 180 | 9000 | 18000 | 9000 | 36000 | 0 | ≥50 | n.r. | [11] |
|  | SiO_2_.phosphate | 200 | 90 | n.s | 180 | 22.5 | 18000 | n.s. | 36000 | **4500** | 1 | ≥50 | n.r. | [11] |
|  | SiO_2_ NM-200 | 189 | 22.5 | 22.5 | 90.0 | n.s. | **4253** | **4253** | 17010 | n.s. | 2 | 1 | 5 | [80] |
|  | SiO_2_ NM-203 | 200 | 90 | 22.5 | 180.0 | n.s. | 18000 | **4500** | 36000 | n.s. | 1 | 1 | 5 | [80] |
| Passive metal oxide and metal sulphate NMs | AlOOH | 105 | 180 | n.s. | n.s | n.s. | 18900 | n.s. | n.s. | n.s. | 0 | (3 ^b^) | (28 ^b^) | [58] |
|  | BaSO_4_ | 41 | n.s. | n.s. | n.s. | n.s. | n.s. | n.s. | n.s. | n.s. | 0 | ≥50 | n.r. | [11] |
|  | Fe_2_O_3_ (hematite) | 98 | n.s. | n.s. | 180 | n.s. | n.s. | n.s. | 8266 | n.s. | 0 | ≥30 | n.r. | [79] |
|  | ZrO_2_.TODA | 117 | 45 | n.s. | 90 | n.s | **5265** | n.s. | 10530 | n.s. | 1 | ≥50 | n.r. | [11] |
|  | ZrO_2_.acrylate | 117 | 70.5 | 141 | 70.5 | 90 | 8249 | 16497 | 8249 | 16497 | 0 | ≥50 | n.r. | [11] |
| Nanosized organic pigments | DPP Orange N | 64 | n.s. | n.s. | n.s | n.s. | n.s. | n.s. | n.s. | n.s. | 0 | ≥30 | n.r. | [79] |
|  | Pigment Blue 15:1 | 53 | 90 | 90 | n.d. ^c^ | n.s. | **4770** | **4770** | n.d. ^c^ | n.s. | 2 | ≥30 | n.r. | [79] |
| Carbonaceous NM | Graphite nanoplatelets | 74 | n.s | 90 | 180 | n.s. | n.s. | 6660 | 13320 | n.s. | 0 | ≥10 | n.r. | [82] |

Footnote to Table S1:

For all parameters, the significant *in vitro* LOAECs (significance as compared to the negative micron-sized benchmark material corundum) are shown, both in mass/volume (µg/mL) and surface area/volume (mm^2^/mL) dose metrics (n.s.: no signficance). The surface area/volume-based values were calculated by multiplying the mass/volume values by the respective NM’s BET surface area. Surface area/volume-based values that undercut the *in vitro* threshold of 6000 mm^2^/mL are provided in bold red. For test material assignment as either active (significant LOAEC <6000 mm^2^/mL, ≥2 of 4 parameters affected) or passive (0 or only 1 parameter affected), the frequency of affected parameters is indicated in the column ‘threshold <6000 mm^2^/mL’. Further, available rat STIS NOAEC values are provided for all test materials (and LOAEC values, if effects were observed). For both the *in vitro* and *in vivo* data, values indicating ‘activity’ are highlighted in yellow, and values indicating ‘passivity’ in green.

a: n.r.: If no effects were observed in the STIS up to the highest tested concentration, no LOAEC was recorded (n.r.).

b: Since AlOOH was tested for 28 days (i.e. 4 consecutive 5-day exposure periods), the NOAEC that Pauluhn [58] recorded for this material (i.e. 3 mg/m^3^) was converted to a 5-day NOAEC by multiplying it by a factor of 4 [94]. Accordingly, the calculated 5-day NOAEC of 12 mg/m^3^ indicates passivity.

c: For technical reasons TNF-α was not determined (n.d.) for Pigment Blue 15:1. However, since significant *in vitro* LOAECs below the threshold were recorded for LDH and GLU, this does not impair its assignment as ‘*in vitro* active’.

d: Numbers in brackets apply to the (short-term) inhalation studies listed in the Reference section.

**Supplementary Information Table S2: Bioactivity of four types of CeO_2_ NMs in rat STISs as compared to cellular effects recorded in the *in vitro* NR8383 AM assay**

|  | ***In vitro* NR8383 alveolar macrophage assay** | | | | | **BALF parameters from STISs** | | | | |
| --- | --- | --- | --- | --- | --- | --- | --- | --- | --- | --- |
|  |  | **LDH** | **GLU** | **TNF-α** | **ROS/H_2_O_2_** |  | **TCC** | **AM** | **PMN** | **Total protein** |
|  | µg/mL | [% PC] | [% PC] | [% standard] | [% PC] | mg/m^3^ | x-fold vehicle control group | | | |
| Al-doped CeO_2_ | 22.5 | 26.0 ± 8.4 | 5.0 ± 0.1 | 64.9 ± 35.2 * | 3.1 ± 0.1 | 0.5 | 1.5 | 1.4 | 14.7 | 1.1 |
|  | 45 | 56.5 ± 11.2 * | 8.6 ± 0.8 | 84.7 ± 14.1 * | 3.2 ± 0.5 | 2.5 | 1.1 | 0.8 | 42.6 | 1.6 |
|  | 90 | 84.1 ± 15.0 * | 19.8 ± 1.3 * | 89.8 ± 9.9 * | 4.2 ± 1.5 | 5 | 2.1 | 0.92 | 156.2 | 3.01 |
|  | 180 | 91.1 ± 14.7 * | 25.0 ± 0.3 * | 88.4 ± 12.6 * | 5.7 ± 0.5 | 10 | 3.3 | 0.7 | 339.9 | 5.4 |
|  |  |  |  |  |  | 25 | 6.5 | 0 | 870.4 | 12.35 |
| CeO_2_ NM-211 | 22.5 | 26.5 ± 8.5 | 6.0 ± 0.1 | 70.8 ± 11.1 * | 0.6 ± 0.9 | 0.5 | 1.1 | 1.1 | 5.4 | 1.1 |
|  | 45 | 38.9 ± 7.8 | 5.7 ± 0.2 | 65.4 ± 13.3 * | 0.3 ± 0.7 | 2.5 | 1.5 | 1.07 | 34.6 | 1.44 |
|  | 90 | 56.5 ± 7.0 * | 6.7 ± 0.2 | 75.0 ± 17.2 * | 2.7 ± 1.2 | 5 | 1.99 | 1.04 | 71 | 1.87 |
|  | 180 | 75.0 ± 7.1 * | 9.9 ± 0.1 * | 85.6 ± 15.6 * | 4.4 ± 1.7 | 10 | 2.96 | 0.98 | 143.9 | 2.73 |
|  |  |  |  |  |  | 25 | 5.9 | 0.8 | 362.5 | 5.3 |
| CeO_2_ NM-212 | 22.5 | 17.9 ± 7.8 | 4.9 ± 0.4 | 63.8 ± 29.4 * | 1.8 ± 0.8 | 0.5 | 1.1 | 1.2 | 4.5 | 1.2 |
|  | 45 | 29.2 ± 12.0 | 6.0 ± 2.2 | 66.7 ± 26.3 * | 1.1 ± 2.1 | 2.5 | 1.28 | 0.98 | 29.14 | 1.51 |
|  | 90 | 45.7 ± 14.6 * | 5.4 ± 0.2 | 76.9 ± 22.0 * | 2.9 ± 0.2 | 5 | 1.5 | 0.7 | 60 | 1.9 |
|  | 180 | 69.0 ± 16.6 * | 9.9 ± 0.8 * | 88.5 ± 10.7 * | 1.8 ± 0.5 | 10 | 2.6 | 0.9 | 129.2 | 2.7 |
|  |  |  |  |  |  | 25 | 5.3 | 0.8 | 332 | 5.1 |
|  | 22.5 | 18.5 ± 5.1 | 4.2 ± 0.2 | 20.5 ± 9.8 | 1.3 ± 0.2 | 0.5 | 1.3 | 1.3 | 4.6 | 0.6 |
|  | 45 | 28.4 ± 3.3 | 4.5 ± 0.1 | 35.8 ± 23.9 * | 1.2 ± 0.5 | 2.5 | 1.3 | 0.4 | 164.1 | 1.4 |
| nano-CeO_2_ | 90 | 45.1 ± 2.1 * | 5.4 ± 0.2 | 89.3 ± 10.6 * | 2.8 ± 1.0 | 5 | 1.83 | 0.65 | 210 | 1.75 |
|  | 180 | 74.3 ± 9.3 * | 9.8 ± 0.5 * | 96.2 ± 5.3 * | 4.7 ± 1.1 | 10 | 2.6 | 0.4 | 386.6 | 2.8 |
|  |  |  |  |  |  | 25 | 4.77 | 0 | 956.38 | 6.13 |

Footnote to Table S2:

Left part: Results from the *in vitro* NR8383 AM assay assessing LDH, GLU, TNF-α, ROS/H_2_O_2_ as recorded in Table 2.

Right part: STIS results, expressed as x-fold to the values recorded for the vehicle control groups 3 days after the final exposure (5-day inhalation exposure, 6 h/day).

Black numbers: measured values as x-fold the untreated control; red numbers: Linearly interpolated values.

TCC: Total cell count; AM: Alveolar macrophages, PMN: polymorphonuclear neutrophils; Total protein concentration (from: Landsiedel *et al.* [11] and Keller *et al*. [111]).
